# Liquid biopsy: current applications and future directions

**DOI:** 10.1186/s43556-026-00533-1

**Published:** 2026-09-07

**Authors:** Yunxiang Zhao, Mengyao Zhang, Yuyan Zhang, Jiahao Bu, Zhibo Liu, Chenran Wang, Li Wang, Xinyu Li, Xianzhi Liu, Weiwei Wang, Jianwei Wei, Liwei Ma

**Affiliations:** 1 Department of Neurosurgery, The First Affiliated Hospital of Zhengzhou University, Zhengzhou, 450052 China; 2 Department of Clinical Laboratory, The First Affiliated Hospital of Zhengzhou University, Zhengzhou, 450052 China; 3 Key Clinical Laboratory of Henan Province, Zhengzhou, 450052 China; 4 Department of Neurology, The First Affiliated Hospital of Zhengzhou University, No.1 Jianshe Dong Road, Zhengzhou, Henan China; 5 Henan Engineering Research Center of Neural Function Detection and Regulation, Zhengzhou, Henan China; 6 Henan Key Laboratory of Cerebrovascular Diseases, (Zhengzhou University), Zhengzhou, China; 7 Department of Pathology, The First Affiliated Hospital of Zhengzhou University, Zhengzhou, 450052 China

**Keywords:** Liquid biopsy, Circulating tumor DNA, Precision medicine, Multi-omics integration, Clinical translation

## Abstract

This review starts with the historical background and biological features to classify mainly the liquid biopsy biomarkers, such as circulating tumor DNA, circulating tumor cells, extracellular vesicles, non-coding RNAs, tumor-educated platelets and tumor-associated antigens, and also summarizes the present classification approaches and advanced detection techniques for each biomarker type. On this basis, the potential clinical uses of liquid biopsy in oncology are discussed in detail, including the early cancer detection, the prediction of the responses to chemotherapy, radiotherapy and immunotherapy, the evaluation of the treatment effects, the monitoring of the residual diseases and the investigation into the tumor heterogeneity and the resistance mechanisms. Moreover, the discussion is extended to other non-oncological fields, involving the reproduction medicine, the transplantation medicine, the neurological disorders, the infectious diseases and various chronic diseases such as the metabolic dysfunction-related liver disease and fibrosis, pulmonary fibrosis, lupus nephritis and renal fibrosis as well as rheumatoid arthritis. Finally, based on the comprehensive summary given above, this review summarizes the current status of the research on liquid biopsy, points out the main obstacles which hinder its wide application in clinic, for example, the absence of standard procedures and the necessity of large-scale prospective validation, and suggests possible future trends like the integration of multi-omics, the use of artificial intelligence in the analysis and the approach towards routine clinical practice.

**Supplementary Information:**

The online version contains supplementary material available at https://doi.org/10.1186/s43556-026-00533-1.

## Introduction

Liquid biopsy, a technique to identify circulating biomarkers in blood and other body fluids, has become an important non-invasive diagnostic means. In 1948, Mandel and Metais discovered the presence of DNA and RNA in human plasma [1]. It wasn't until the 1970 s to 1990 s that the relationship between circulating nucleic acids and cancer was confirmed, showing that the DNA from tumors had some mutations [2, 3]. This discovery, together with the identification of circulating tumor cells [4], extracellular vesicles [5], and non-coding RNAs [6, 7], forms the basis of liquid biopsy. Compared to the traditional tissue biopsy, liquid biopsy is less invasive, more reproducible and can reflect the heterogeneity of the disease. The progress of digital polymerase chain reaction, next generation sequencing and biosensors has promoted its use in clinics. With the rapid development in this field, a comprehensive review is necessary covering the historical background, classification of markers, working principles and clinical applications. 

This paper aims to summarize the main liquid biopsy markers, introduce the detection methods and clinical applications in oncology as well as in non-oncological diseases. In oncology, it mainly concentrates on the early diagnosis, prediction of treatment response, monitoring of resistance and the study of heterogeneity. Besides oncology, we also take into account reproductive medicine, transplantation, neurological disorders and infectious diseases. The structure of this paper comprises: first, classifying the major liquid biopsy markers and tracing their discovery and research history; second, comparing the basic detection techniques according to their principles, sensitivity and clinical values; third, summarizing the clinical applications of liquid biopsy during the cancer treatment process; fourth, exploring possible new uses in non-oncological diseases; fifth, discussing the existing problems like standardization, biological noise and the need for prospective validation and suggesting some research directions such as multi-omics integration and intelligent analysis, to offer clear guidance for researchers and doctors in the personalized medicine era.

## Classification of liquid biopsy biomarkers

### Circulating tumor DNA/RNA (ctDNA/RNA)

Cell-free DNA (cfDNA) or cell-free RNA were first detected by Mandel and Metais in 1947 by means of chemical methods and were found in a free and precipitated form [8]. Although the polymerase chain reaction (PCR) and sequencing techniques were not available at that time [1], their finding shows the existence of extracellular DNA/RNA in the blood. At first, these substances were usually referred to as cell-free DNA (cfDNA) or cell-free RNA. It was not until the beginning of the twenty-first century, with the development of liquid biopsy technique, that cfDNA and cfRNA were widely used for the specific investigation of these circulating nucleic acids. In ordinary persons, cfDNA and cfRNA are mainly originated from normal physiological activities such as programmed cell death (apoptosis) and cellular regeneration [9].

The connection between cfDNA and cancer was first established in 1977 by Leon et al., who reported significantly higher cfDNA concentrations in cancer patients compared to healthy controls, suggesting its potential as a disease indicator [2]. However, it was not until 1994 that the tumor origin of cfDNA was confirmed, when mutated *RAS* gene fragments were detected in the peripheral blood of patients with hematological malignancies, while no such mutations were found in healthy donors [3, 10]. This evidence demonstrated that DNA released by tumor cells carries tumor-specific genetic alterations, leading to the designation of this fraction as circulating tumor DNA (ctDNA) and its RNA counterpart as ctRNA. Today, driven by rapid advances in quantitative technologies, ctDNA/ctRNA analysis has demonstrated broad clinical utility, including monitoring minimal residual disease, predicting recurrence risk, enabling early cancer detection, facilitating real-time treatment response assessment, and exploring mechanisms of drug resistance [11–13].

### Circulating tumor cells (CTCs)

Circulating tumor cells (CTCs) are intact tumor cells that shed from primary or metastatic solid tumors into the bloodstream, either spontaneously or as a result of diagnostic or therapeutic interventions. The first documented observation of CTCs dates back to 1869, when Australian pathologist Thomas Ashworth identified cells morphologically similar to those of the original tumor in the blood of a patient who had died from cancer. He proposed that these cells might originate from the tumor and disseminate via the bloodstream, potentially serving as the basis for metastasis [4]. However, due to the technological limitations of the era, in-depth study of these cells was not feasible.

Between the 1950 s and 1970 s, advances in cytopathology enabled further exploration. Numerous case reports, based on light microscopy examination of blood smears from patients with advanced cancer, described the presence of CTCs in peripheral blood, particularly in those with extensive metastasis [14, 15]. Initial attempts to develop routine cytological methods for CTC detection reported high positivity rates in cancer patients. However, these findings were soon attributed to false positives, leading researchers to abandon conventional cytological approaches for CTC identification [16].

An advance has been achieved in immunocytochemistry during the 1990 s and early 2000s. Scientists used antibodies against epithelial cell-specific markers, such as epithelial cell adhesion molecule (EpCAM) and cytokeratins (CKs), which were labeled with magnetic beads to specifically separate CTCs from epithelial tumors [17]. This immunomagnetic enrichment method can improve the efficiency and specificity of CTC purification, allowing for accurate counting and further statistical analysis.

In 2004, the clinical utility of CTC enumeration was solidified with U.S. Food and Drug Administration (FDA) approval of the CellSearch system for prognostic assessment in metastatic breast, colorectal, and prostate cancer. As the first standardized and reproducible commercial platform for CTC detection, CellSearch facilitated numerous clinical studies that established elevated CTC counts as an independent prognostic factor for poorer outcomes in patients with metastatic cancers [18–21]. Beyond enumeration, contemporary CTC research has expanded into functional and molecular characterization. Emerging applications include single-cell genomic and transcriptomic analysis, the establishment of patient-derived organoid cultures from CTCs, and in-depth profiling of cell-surface proteins, all of which offer new opportunities and challenges for personalized cancer therapy [22–24].

### Extracellular vesicles (EVs)

The history of extracellular vesicles (EVs) dates back to the 1940 s, when "microparticles" or "vesicle-like structures" were observed in plasma and other body fluids using electron microscopy. At the time, however, they were largely dismissed as cellular debris or experimental artifacts [25]. A pivotal shift occurred in the 1980s. While studying the maturation of reticulocytes into erythrocytes, Johnstone's group discovered that these cells released small, membrane-bound vesicles containing transferrin receptors—proteins no longer needed in the mature cell [5]. In 1987, Johnstone termed this specific type of vesicle, which is formed through endocytosis and secreted extracellularly, an "exosome" [5]. Initially, exosomes were thought to function merely as a cellular waste disposal system for eliminating unwanted proteins [5, 26].

The first report on the biological function of exosomes was given in 1996, when Raposo and his colleagues showed that exosomes from B lymphocytes have MHC class II molecules on their surface and could activating T cells in vitro. This important discovery suggested that exosomes might play a role in cell-to-cell communication [27]. In 2007, the research of Lötvall and Breakefield separately discovered that in addition to proteins, exosomes contain functional mRNAs and microRNAs (miRNAs) as well. They definitely proved that these RNAs could be transferred to the target cells and affect their functions [28, 29]. Thus, exosomes are considered to carry genetic information which can promote gene transfer between cells, indicating possible uses in disease diagnosis and treatment, especially in oncology.

At present, the study on exosomes is developing fast and they are being used as promising markers, carriers for treatment and even new drug targets [30]. However, some problems still exist. The variety of exosome types and the absence of standardized techniques for isolation and characterization hinder their application in clinical practice. It is an urgent task to establish reliable and reproducible methods for the separation, identification and examination of exosomes in this area [30–32].

### Non-coding RNAs in liquid biopsy: miRNAs and lncRNAs

Beyond protein-coding genes, non-coding RNAs (ncRNAs) have been found to be important in the liquid biopsy field. Among them, microRNAs (miRNAs) and long non-coding RNAs (lncRNAs) have received special attention because of their regulatory functions and existence in biofluids, usually contained in extracellular vesicles (EVs) or linked to circulating tumor cells (CTCs).

#### MicroRNAs (miRNAs)

MicroRNAs are a class of short, non-coding RNA molecules, typically 18–25 nucleotides in length, that function as key post-transcriptional regulators of gene expression [33]. The first miRNA was discovered in 1993 by the laboratories of Victor Ambros and Gary Ruvkun while studying developmental timing mutants in *C. elegans*. Although this finding was published in *Science*, it was initially considered a nematode-specific phenomenon and did not immediately attract broad scientific interest [6, 33, 34]. This perspective shifted dramatically in 2000, when Ruvkun's laboratory identified a second miRNA, let-7, also in nematodes. Critically, they demonstrated that let-7 was evolutionarily conserved across species, including flies, mice, and humans, suggesting that miRNAs represent a ubiquitous and fundamental mechanism of gene regulation rather than a nematode peculiarity [7].

The discovery of let-7 catalyzed the field, leading to a surge of miRNA-related publications in 2001 and the official coining of the term "microRNA." Subsequent research has revealed complex regulatory networks involving miRNAs and their mRNA targets, with dysregulated miRNA expression implicated in nearly all major human diseases, including cancer, cardiovascular disorders, and neurodegenerative conditions [35–37]. The high stability of miRNAs in blood, coupled with their tissue specificity and ability to reflect real-time physiological states, has positioned them as promising candidates for non-invasive disease diagnosis and prognosis [38]. Recent advances have focused on several key areas: (i) elucidating the role of EVs as critical carriers for intercellular miRNA communication [28]; (ii) improving computational tools (e.g., TargetScan) and databases (e.g., miRBase) for target prediction and annotation [39, 40]; and (iii) shifting from single-molecule analyses to systems-level approaches, such as investigating miRNAs within competing endogenous RNA (ceRNA) networks. Ultimately, these efforts aim to translate miRNA biology into clinical applications, including the development of circulating miRNAs as diagnostic biomarkers and their exploration as novel therapeutic targets and agents [41].

#### Long non-coding RNAs (lncRNAs)

Long non-coding RNAs (lncRNAs) are defined as RNA transcripts exceeding 200 nucleotides in length that lack protein-coding potential. Once dismissed as transcriptional "noise" or "junk DNA," lncRNAs are now recognized as crucial regulators of gene expression. Early hints of their importance predate the completion of the Human Genome Project. For instance, *H19* (discovered in 1984) and *Air* (discovered in the 1990 s) were identified as long transcripts involved in genomic imprinting [42, 43]. Although initially classified as "imprinted genes," the functional roles of their non-coding transcripts remained obscure for many years [42]. Subsequent studies revealed that the promoter region of *H19* is critical for imprinting regulation, and the act of *Air* RNA transcription itself is essential for cis-silencing of neighboring genes [43, 44]. These pioneering observations laid the groundwork for modern lncRNA research.

A paradigm shift occurred with the ENCODE project, launched in 2003. Through high-throughput sequencing, ENCODE revealed that over 75% of the human genome is transcribed, yet less than 2% encodes proteins [45]. This discovery fundamentally overturned the traditional view of the genome, demonstrating that regions once considered "junk DNA" constitute a vast and unexplored "non-coding RNA universe" rich with lncRNAs [46]. With the cataloging of lncRNAs underway, the field's focus shifted to functional characterization. Loss-of-function experiments, evolving from early RNA interference (RNAi) to modern CRISPR-based screens, have provided indispensable causal evidence for lncRNA functions [47]. Today, lncRNA research is a mature and highly active field, with increasing emphasis on mechanistic dissection and clinical translation. Dysregulated lncRNA expression has been documented in numerous diseases. In oncology, lncRNAs such as *H19*, *PVT1*, *NEAT1*, and *MALAT1* promote tumor progression by modulating proliferation, metastasis, immune evasion, and metabolic reprogramming [48–51]. In neurodegenerative disorders like Alzheimer's and Parkinson's diseases, lncRNAs including *MALAT1* and *BDNF-AS* are implicated in neuronal apoptosis and inflammatory responses [52–54]. In cardiovascular disease, lncRNAs such as *MHRT* and *CHRF* are closely associated with cardiac hypertrophy and heart failure [55, 56].

Despite the promising therapeutic potential of targeting lncRNAs, significant challenges impede clinical translation. First, precise elucidation of core lncRNA functions within complex regulatory networks is essential to avoid off-target effects. Second, specificity and safety remain major technical hurdles; antisense oligonucleotides (ASOs) and siRNAs require highly specific recognition and delivery systems to minimize off-target effects [57, 58]. Concurrently, novel drug development paradigms are needed, such as designing small molecule inhibitors based on lncRNA three-dimensional structures [59]. Future progress will likely depend on innovative strategies, including personalized approaches like utilizing patient-derived exosomes as delivery vehicles to achieve precision targeting [60].

### Other circulating biomarkers

Besides the recognized markers mentioned before, some other blood substances have also been found as promising methods in liquid biopsy. In this part, we will discuss two of them: tumor-educated platelets (TEPs) and tumor-associated antigen autoantibodies (TAAbs), both of which indicate tumor-host interactions and provide special possibilities for diagnosis and surveillance.

#### Tumor-educated platelets (TEPs)

Tumor-educated platelets (TEPs) refer to platelets that have been phenotypically and functionally modified by tumor cells through direct or indirect interactions within the tumor microenvironment, leading to alterations in their mRNA expression profiles, proteome, and functional behavior [61]. The association between tumors and platelets dates back to 1872, when Leopold Riess first observed thrombocytosis in cancer patients, although the underlying mechanism and significance remained unclear at the time [62]. Over the following century, advances in laboratory techniques expanded our understanding of platelet functions beyond hemostasis and coagulation. Approximately 50 years ago, the scientific community began to recognize the link between elevated platelet counts and cancer progression, and discovered that platelet-mediated thrombosis might facilitate tumor cell metastasis [63, 64].

From the late twentieth century to the early 2000 s, accumulating evidence revealed complex bidirectional interactions between platelets and tumor cells. On one hand, tumor cells can "educate" platelets by transferring biomolecules (e.g., RNA) through direct contact or exosome release, altering platelet RNA splicing patterns [28, 65]. On the other hand, educated platelets reciprocally influence tumor behavior by releasing pro-angiogenic factors such as vascular endothelial growth factor (VEGF) and platelet-derived growth factor (PDGF), or by shielding circulating tumor cells (CTCs) from immune surveillance, thereby promoting metastasis [61, 66]. Importantly, despite being anucleate, platelets contain precursor mRNAs, functional spliceosomes, and translational machinery, enabling them to perform pre-mRNA splicing and protein synthesis in response to external stimuli, including tumor-derived signals [67]. Additionally, platelet-derived microparticles released upon activation have been implicated in tumor progression, with some studies suggesting context-dependent pro- or anti-tumorigenic effects [65].

The term "tumor-educated platelets" gained traction around 2010 to describe this tumor-modified platelet state. A pivotal technical advance came with the development of the ThromboSeq platform, an RNA sequencing-based method capable of detecting tumor-associated RNA splicing patterns from minute quantities of platelet RNA. Coupled with machine learning algorithms, ThromboSeq enables classification of RNA profiles across different disease states and even prediction of primary tumor origin [68]. Subsequently, TEP research has entered a phase of extensive validation and expansion, with multiple studies evaluating TEP-based diagnostics in large cohorts and exploring their utility in treatment monitoring [69, 70].

Despite their promise, TEPs face several challenges prior to clinical implementation. Non-malignant conditions such as inflammation and cardiovascular disease can also alter platelet RNA profiles, potentially confounding tumor-specific signals [68]. Moreover, the precise molecular mechanisms underlying platelet education remain incompletely understood. From a translational perspective, two core hurdles persist. First, economic and technical feasibility: current reliance on RNA sequencing entails high costs, non-negligible error rates, and complex data analysis, limiting accessibility in routine clinical settings [71]. Second, the breadth and depth of clinical evidence: most studies to date have been retrospective and based on limited cohort sizes, raising concerns about generalizability. Rigorous, large-scale prospective studies encompassing diverse populations are urgently needed to establish the clinical utility of TEPs.

#### Tumor-associated antigen autoantibodies (TAAbs)

Tumor-associated antigen autoantibodies (TAAbs) are antibodies produced by the host immune system specifically against tumor-associated antigens (TAAs). As early as the 1960 s and 1970 s, researchers using immunofluorescence techniques detected antibodies capable of binding to autologous tumor cells in sera from patients with melanoma, neuroblastoma, and selected other cancers [72, 73]. These seminal observations demonstrated for the first time that the cancer patient's immune system could recognize and mount a humoral response against tumor cells, challenging the prevailing dogma that the immune system targeted only "non-self" and not "self." This laid the conceptual foundation for "tumor antigens."

Technical limitations initially precluded identification of the specific antigenic targets of these antibodies. The advent of monoclonal antibody technology and Western blotting in the 1980 s enabled scientists to resolve individual protein bands from complex cellular mixtures. In 1982, autoantibodies against the p53 tumor suppressor protein were first identified in sera from breast cancer patients [74]. Subsequently, autoantibodies against a range of TAAs—including HER2/neu, NY-ESO-1, and members of the MAGE family—were discovered [75–77]. However, these early studies were largely restricted to single antigen–antibody pairs, providing an incomplete picture of the humoral immune response in cancer.

A major breakthrough occurred in 1995 with the development of SEREX (serological identification of antigens by recombinant expression cloning) by Sahin and colleagues [78]. This method, which involves screening tumor cDNA libraries with autologous patient serum, enabled systematic identification of numerous TAAs recognized by the humoral immune system. Concurrently, proteomics-based approaches, including two-dimensional electrophoresis coupled with mass spectrometry and serum proteomics analysis, provided complementary high-throughput avenues for TAAb discovery [79]. These technological advances facilitated the development of multi-plex assays capable of simultaneously detecting panels of TAAbs, significantly enhancing diagnostic accuracy and specificity.

Contemporary TAAb research is increasingly oriented toward clinical application. High-throughput protein microarrays now enable simultaneous detection of antibodies against hundreds of TAAs from minute serum volumes [80]. The resulting high-dimensional data have necessitated sophisticated bioinformatic approaches, with machine learning algorithms such as support vector machines (SVM) and random forests being systematically applied and compared. Random forests, in particular, have demonstrated superior performance in handling high-dimensional, non-linear data and distinguishing cancer patients from healthy controls, as well as differentiating among cancer types. Because TAAbs may arise at very early stages of tumorigenesis, they are considered promising candidates for early cancer detection within the liquid biopsy paradigm [81]. Several biotechnology companies are currently developing cancer screening products based on TAAb detection, with panels for lung, liver, and ovarian cancer entering clinical trials or being offered as laboratory-developed tests.

**Fig. 1:**
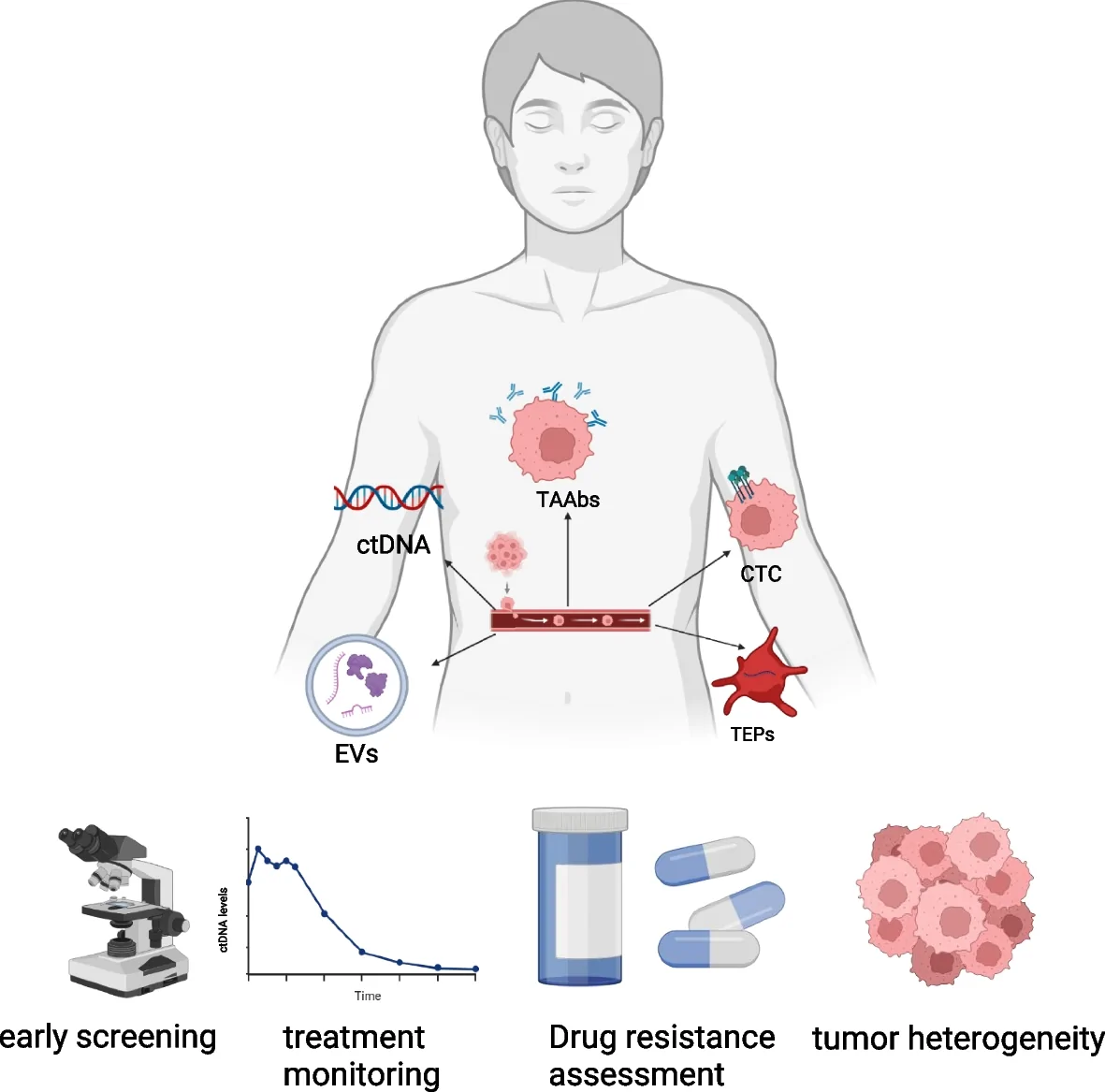
Liquid biopsy enables dynamic management of tumors throughout the tumor cycle by analyzing biomarkers from multiple sources in the blood. This schematic illustrates how various blood-derived biomarkers—including circulating tumor DNA (ctDNA), circulating tumor cells (CTCs), extracellular vesicles (EVs), and non-coding RNAs—are released from primary tumors and metastatic sites into the bloodstream. These analytes can be captured and analyzed through liquid biopsy to enable early cancer screening, treatment response monitoring, minimal residual disease (MRD) surveillance, and analysis of tumor heterogeneity and acquired resistance

Nevertheless, some problems exist in TAAb-based diagnosis. It is essential to standardize the detection procedures and determine the universal positive cutoff values for its extensive clinical use. It is suggested that in the future, combining TAAb with other liquid biopsy markers, like ctDNA and exosomal biomarkers, in multi-omics diagnostic models may improve the sensitivity and specificity. We compared the key characteristics of these biomarkers, including their sources, detection methods, application areas, advantages, and limitations (Table 1), while Fig. 1 provides a conceptual overview of how liquid biopsy integrates multiple blood-derived analytes to enable dynamic tumor monitoring across the disease continuum.

**Table 1:** Comparison of key liquid biopsy biomarkers

Biomarker	Primary Sources	Core Detection Methods	Main Application Areas	Advantages	Limitations	Reference
Circulating Tumor DNA (ctDNA)	Apoptosis, necrosis, or active secretion of tumor cells	ddPCR, NGS	Early screening, MRD monitoring, treatment response assessment, resistance mechanism analysis	Non-invasive; represents whole tumor genome; enables dynamic monitoring	Low abundance in early-stage disease; requires highly sensitive technologies	[82]
Circulating Tumor Cells (CTCs)	Intact tumor cells shed from primary or metastatic sites	CellSearch, flow cytometry, single-cell sequencing	Prognostic stratification, metastasis research, ex vivo culture for functional studies	Provides intact viable cells for phenotypic and genomic analysis	Extreme rarity in blood; technically challenging isolation and enrichment	[83]
Extracellular Vesicles (EVs)	Actively secreted by various cell types, including tumor cells	Ultracentrifugation, nano-flow cytometry, immunocapture	Disease diagnosis, therapeutic carrier development, intercellular communication studies	Carry diverse molecular cargo (proteins, RNAs); high stability in biofluids	Heterogeneous populations; lack of standardized isolation protocols	[84, 85]
miRNA/lncRNA	Encapsulated in EVs/CTCs or bound to protein complexes	Microarray, small RNA sequencing, qPCR	Non-invasive diagnostic/prognostic biomarkers; therapeutic targets	High stability; tissue/cancer-type specificity; key regulatory functions	Complex regulatory networks; functional validation challenging	[57–59]
Tumor-Educated Platelets (TEPs)	Platelets reprogrammed by tumor-derived signals	RNA sequencing (e.g., ThromboSeq)	Early cancer detection, tumor type classification	Easily accessible sample; rich RNA profile reflecting tumor presence	Potential interference from non-malignant conditions (e.g., inflammation)	[68, 70]
Tumor-Associated Antigen Autoantibodies (TAAbs)	Produced by the host immune system against tumor antigens	Protein microarrays, SEREX, ELISA	Early cancer screening, diagnostic adjuncts	Immuno-amplification enables detection even at low antigen levels; early emergence	Moderate specificity; often requires panels of multiple TAAbs	[78, 80, 81]

## Detection methods for liquid biopsy markers

### PCR-derived techniques

Conventional PCR techniques face limitations in detecting mutations with low allele frequencies. Specifically, the detection limit for mutant allele frequency (MAF) using standard methods is typically around 20%, due to background noise such as PCR errors and sequencing artifacts [86]. In the context of liquid biopsy, where circulating tumor DNA (ctDNA) often constitutes less than 0.1% of total cell-free DNA—particularly in early-stage cancer or minimal residual disease (MRD)—this level of sensitivity is insufficient for reliable clinical detection. To address this, several PCR-derived technologies have been developed, including quantitative PCR (qPCR)-based methods and digital PCR. Among these, Amplification Refractory Mutation System PCR (ARMS-PCR) and droplet digital PCR (ddPCR) have emerged as widely adopted tools for sensitive mutation detection in liquid biopsy applications.

ARMS-PCR(also known as allele-specific PCR) is a method designed to detect known point mutations [87]. The principle leverages the inherent property of DNA polymerases, such as Taq polymerase, which require precise complementarity at the 3' end of the primer for efficient extension. A mismatch at the terminal 3' base between the primer and the template dramatically reduces or abolishes amplification. ARMS-PCR exploits this by designing mutation-specific primers whose 3' end is perfectly complementary to the mutant sequence but mismatched with the wild-type template. Thus, amplification occurs preferentially in the presence of the mutant allele. To further enhance specificity, an additional, deliberate mismatch is often introduced near the 3' end (typically at the second or third base from the terminus) [88]. This secondary mismatch destabilizes the primer-template duplex, particularly when the 3' end is already mismatched (as with wild-type templates), thereby minimizing non-specific amplification. Due to its simplicity and cost-effectiveness, ARMS-PCR was once the mainstream method for detecting known hotspot mutations, such as those in *EGFR* and *KRAS*. However, its sensitivity is relatively limited, and it can only detect pre-defined mutations [89, 90]. In liquid biopsy, ARMS-PCR is primarily applied to detect known clinically actionable hotspot mutations in ctDNA, such as EGFR mutations (exon 19 deletions, L858R, T790M) in non-small cell lung cancer and KRAS mutations in colorectal cancer [91–94]. Its main advantage in this setting is rapid turnaround time (results within hours) and low cost, making it suitable for initial screening of advanced-stage cancer patients where ctDNA abundance is relatively high. However, due to its limited sensitivity (typically requiring MAF ≥ 1–5%), ARMS-PCR may fail to detect mutations in early-stage cancers or post-treatment MRD samples where ctDNA levels are extremely low [91].

Droplet digital PCR (ddPCR) represents a paradigm shift from traditional end-point or real-time PCR. Instead of relying on cycle threshold (Ct) values for quantification—which can be influenced by PCR efficiency and sample inhibitors—ddPCR achieves absolute quantification through sample partitioning. The PCR reaction mixture is partitioned into tens of thousands of nanoliter-sized water-in-oil droplets using a droplet generator. Each droplet contains either zero, one, or multiple target DNA molecules. Following PCR amplification to endpoint, droplets are analyzed for fluorescence, allowing classification as positive or negative. The proportion of positive droplets is then fitted to a Poisson distribution to calculate the absolute concentration of target molecules in the original sample without requiring standard curves [95]. This approach confers several advantages: ultra-high sensitivity (detecting MAF as low as 0.001–0.1%), tolerance to PCR inhibitors, and absolute quantification capability [96]. However, it is important to distinguish between technical sensitivity and clinically actionable sensitivity. The 0.001% limit of detection represents the optimal performance achievable under ideal conditions using purified synthetic DNA or well-characterized cell line samples. In real-world clinical practice, biological noise—including clonal hematopoiesis (CHIP)-derived mutations, cell-free DNA shed from normal tissues, and the inherently fragmented nature of ctDNA—imposes a practical detection floor, typically around 0.1% for reliable clinical decision-making in early cancer detection. Therefore, while ddPCR offers exceptional technical sensitivity, the biological background in patient samples remains a critical limiting factor that distinguishes "detectable" from "clinically actionable" mutations. Despite its advantages, ddPCR is limited to detecting a small number (typically 2–4) of predetermined mutations per reaction and requires prior knowledge of target sequences for probe design [97, 98]. In liquid biopsy, ddPCR has become an indispensable tool for applications requiring the highest sensitivity and precise quantification [97, 98]. Key applications include: minimal residual disease (MRD) monitoring after curative-intent surgery, where detecting residual ctDNA at ultra-low levels can predict relapse months before radiographic evidence [11]; longitudinal tracking of treatment response, where serial ctDNA quantification provides early readouts of therapeutic efficacy [13]; and detection of emerging resistance mutations (e.g., EGFR T790M in NSCLC patients progressing on first-generation TKIs) at low abundance, enabling timely clinical intervention [99]. The absolute quantification capability of ddPCR is particularly valuable for monitoring ctDNA dynamics over time, as it eliminates inter-run variability inherent to qPCR-based methods. Despite its advantages, ddPCR is limited to detecting a small number (typically 2–4) of predetermined mutations per reaction and requires prior knowledge of target sequences for probe design [97, 98]. This makes ddPCR well-suited for monitoring known patient-specific mutations but unsuitable for discovery-based applications or comprehensive genomic profiling, which instead require next-generation sequencing approaches. In summary, ARMS-PCR and ddPCR differ substantially in sensitivity, throughput, and quantification capability (Table 2).

**Table 2:** Comparison of ARMS-PCR and Droplet Digital PCR (ddPCR) for liquid biopsy applications

Feature	ARMS-PCR	Droplet Digital PCR (ddPCR)	References
Technical Principle	Allele-specific amplification using primers with 3'-end complementarity to mutant sequences; wild-type template amplification is blocked by terminal or near-terminal mismatches	Sample partitioning into thousands of nanoliter-sized droplets; end-point PCR amplification followed by Poisson statistical analysis for absolute quantification	[87]
Nature of Detection	Indirect, endpoint or real-time detection based on fluorescence signal; relative quantification using Cq values	Direct, digital readout based on counting positive vs. negative droplets	[87, 95, 96]
Sensitivity (MAF)	Low to moderate (typically 1–5% mutant allele frequency)	Ultra-high (as low as 0.001–0.1% mutant allele frequency)	[91]
Quantification Method	Semi-quantitative or relative quantification; requires standard curves for absolute quantitation	Absolute quantification without standard curves; direct concentration measurement	[97, 98]
Key Advantages	Rapid turnaround; low cost; simple workflow; well-established technology; minimal equipment requirements	Ultra-high sensitivity; precise quantification; tolerance to PCR inhibitors; high reproducibility; ideal for rare mutation detection	[89, 96, 100]
Major Limitations	Limited sensitivity; cannot detect unknown mutations; restricted multiplexing capability	Low throughput per run; cannot detect unknown mutations; requires specialized equipment; higher cost per sample	[90, 91, 101]
Liquid Biopsy Applications	Rapid screening for known hotspot mutations (e.g., *EGFR*, *KRAS*, *BRAF*) • Complementary tool in resource-limited settings • Initial triage before reflex NGS testing	• Ultra-low MAF mutation detection • Treatment efficacy monitoring • Minimal residual disease (MRD) surveillance • Validation and orthogonal confirmation of NGS findings • Analysis of samples with inhibitors or degraded DNA	[98, 100–103]

This table compares two PCR-based methods optimized for detecting circulating tumor DNA (ctDNA) mutations. ARMS-PCR (Amplification Refractory Mutation System PCR) uses allele-specific primers to detect known hotspot mutations, offering rapid turnaround and low cost, which makes it suitable for initial screening in advanced-stage cancers. ddPCR (droplet digital PCR) partitions the reaction into thousands of nanoliter-sized droplets for absolute quantification, providing ultra-high sensitivity (MAF as low as 0.001–0.1%), tolerance to inhibitors, and high reproducibility. Consequently, ddPCR is particularly valuable for minimal residual disease (MRD) monitoring, longitudinal treatment tracking, and detection of emerging resistance mutations. The table details the technical principle, detection type, sensitivity (MAF), quantification method, key advantages, major limitations, and representative liquid biopsy applications for both methods

### Next-generation sequencing (NGS)

Before the appearance of next-generation sequencing (NGS), Sanger sequencing was mainly used for DNA sequencing. But its disadvantages such as low output rate, high expense and slow speed resulted in some difficulties, which made large-scale genomic applications unattainable for regular research and medical use [103]. The introduction of NGS was a technological revolution due to both the scientific necessity and commercial advancement, changing sequencing from a 'one reaction, one read' concept into a 'millions of parallel sequencing reactions.'

The conceptual foundation for NGS can be traced to the 1990 s, when Lynx Therapeutics developed Massively Parallel Signature Sequencing (MPSS). This technology employed bead-based capture of single DNA molecules, emulsion PCR amplification, and ligation-based sequencing on assembled bead arrays. Although MPSS was technically complex, suffered from short read lengths and high costs, and ultimately failed to achieve commercial success, it introduced several revolutionary concepts—massively parallel processing, emulsion PCR, and ligation-based sequencing—that became foundational for later platforms [104]. The first commercially successful NGS platform was launched in 2005 by Roche's 454 Life Sciences. Building on MPSS concepts, the 454 system utilized emulsion PCR amplification on microbeads followed by pyrosequencing, which detected nucleotide incorporation through chemiluminescent signals. This platform increased sequencing throughput approximately 100-fold compared to Sanger methods, marking a pivotal moment in genomics [105]. In 2006, Illumina (formerly Solexa) introduced a fundamentally different approach: bridge amplification on flow cell surfaces to generate clonal clusters, followed by sequencing-by-synthesis using reversible terminator chemistry. With dramatically higher throughput and lower costs, Illumina rapidly captured the market and has since become the dominant player, holding over 90% of the global sequencing market share [105].

In 2007, Applied Biosystems (ABI) introduced the SOLiD (Sequencing by Oligonucleotide Ligation and Detection) system. This platform employed emulsion PCR on beads followed by ligation-based sequencing using fluorescently labeled oligonucleotides. While theoretically offering the highest accuracy due to its two-base encoding mechanism, SOLiD was hampered by technical complexity, short read lengths, and long run times, ultimately losing market competitiveness [106].

**Fig. 2:**
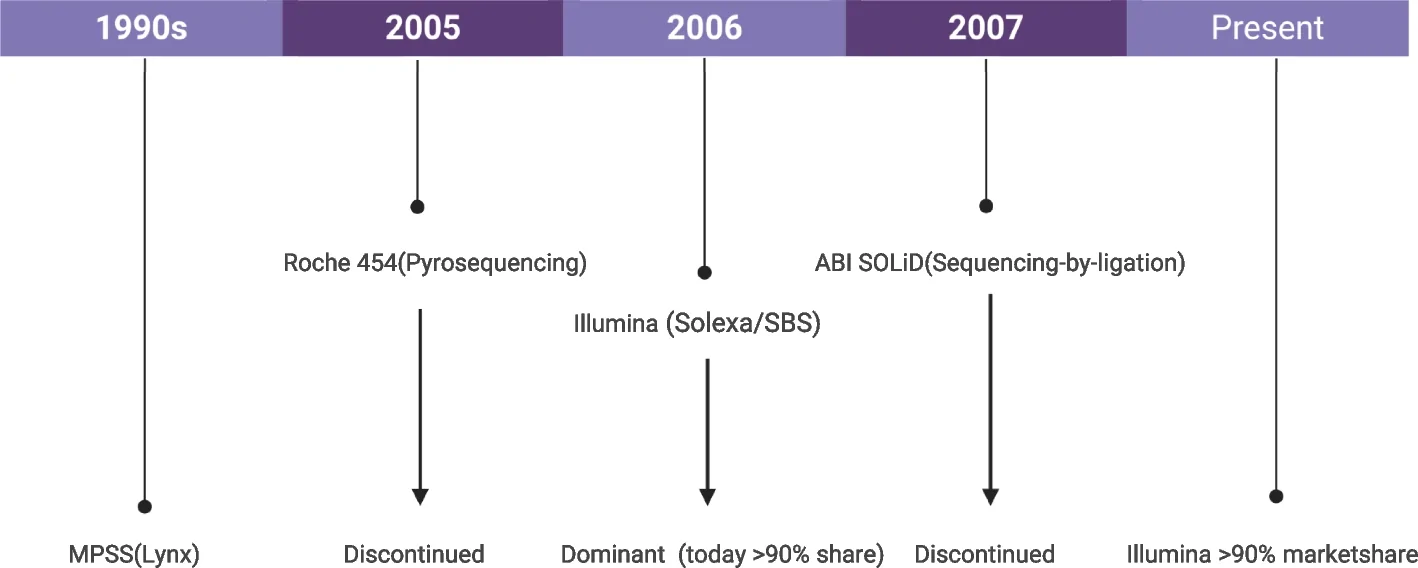
Timeline of early NGS platform development. MPSS (1990s) introduced key concepts but failed commercially. Roche 454 (2005) was the first successful NGS platform but was later discontinued. Illumina (2006) became the market leader due to its high throughput and low cost. ABI SOLiD (2007) offered high theoretical accuracy but lost the competition due to technical complexity and short read lengths

Figure 2 presents a timeline summarizing the development and commercial outcomes of these early NGS platforms, from the conceptual breakthrough of MPSS in the 1990 s to Illumina's eventual market domination. In the context of liquid biopsy, NGS has become indispensable because it addresses a fundamental limitation of PCR-based methods: the ability to discover unknown mutations and profile multiple genomic alterations simultaneously from limited amounts of circulating tumor DNA (ctDNA) [107]. Unlike ARMS-PCR or ddPCR, which can only detect pre-defined mutations, NGS enables unbiased comprehensive genomic profiling—a critical capability given that resistance mechanisms and tumor evolution often involve unexpected mutations or complex genomic rearrangements. Key liquid biopsy applications of NGS include comprehensive genomic profiling of ctDNA, which enables simultaneous detection of single nucleotide variants, insertions/deletions, copy number alterations, and gene fusions from a single blood sample, allowing identification of actionable alterations across dozens or hundreds of cancer-related genes simultaneously in patients with advanced cancers where tissue biopsy is insufficient or infeasible [107]. NGS also facilitates blood-based tumor mutational burden and microsatellite instability assessment, as sequencing broad panels of genes can estimate the total number of somatic mutations in ctDNA to predict response to immune checkpoint inhibitors [108, 109], while analysis of microsatellite loci enables non-invasive determination of MSI status to identify patients likely to benefit from immunotherapy [110]. Furthermore, NGS-based analysis of ctDNA methylation patterns and fragmentation profiles has enabled the development of multi-cancer early detection assays that can screen for dozens of cancer types from a single blood draw with high accuracy in predicting tissue of origin [111, 112], leveraging NGS's ability to interrogate millions of genomic loci simultaneously—a scale unattainable with targeted PCR methods. Finally, longitudinal NGS analysis of ctDNA during treatment can reveal the emergence of novel resistance mutations that were not pre-specified in a PCR panel, enabling discovery of unexpected mechanisms of acquired resistance that is essential for understanding treatment failure and developing next-generation therapies [113].While the first-generation NGS platforms laid the foundation for high-throughput genomics, the sequencing landscape has since evolved dramatically(Table 3), which provides a comparison of contemporary high-throughput sequencing platforms available in 2024–2026, including both short-read workhorses and emerging long-read technologies that are increasingly applied to liquid biopsy applications such as structural variant detection and comprehensive genomic profiling.

**Table 3:** Comparison of contemporary high-throughput sequencing platforms (2024–2026)

Feature	Illumina NovaSeq X Plus	Element AVITI	MGI DNBSEQ-T20 × 2	Ultima UG 100	PacBio Revio (Long-read)	Oxford Nanopore PromethION 2 (Long-read)
Sequencing Principle	SBS (Reversible terminators)	SBS (Avidity base chemistry)	DNBSEQ (Rolling circle amplification)	SBS (Native-like nucleotides)	SMRT sequencing	Nanopore-based
Max Output per Run	~ 16 Tb (dual flow cell)	~ 1 Tb	~ 18–72 Tb	~ 9 Tb	~ 360 Gb (HiFi)	~ 58 Tb (24-h run)
Read Length	2 × 150 bp	2 × 150 bp	2 × 150 to 2 × 300 bp	2 × 300 bp	~ 15–25 kb (HiFi)	Up to 4 Mb (ultra-long)
Time per Run	~ 24–48 h	~ 24 h	~ 48–72 h	~ 20 h	~ 30 h	Variable (real-time)
Cost per Gb	~ $2	~ $5–7	~ $1–2 (lowest)	~ $1–3	~ $10–15	~ $5–10
Key Advantage	Established ecosystem, highest throughput	Lower cost, smaller footprint	Extremely low cost per Gb	Rapid turnaround, low cost	Long reads, high accuracy	Ultra-long reads, real-time, portable
Primary Application in LB	Comprehensive genomic profiling, MCED	Clinical labs with moderate volume	Population-scale screening	Rapid point-of-care deployment	Structural variants, fusions	Real-time pathogen detection, structural variants
References	[114]	[115]	[116]	[117]	[118]	[119]

Driven by continuous technological innovation, high-throughput sequencing is evolving toward greater speed, comprehensiveness, accuracy, and accessibility. Recent advances have focused on three major directions: ultrafast genomics, long-read sequencing technologies, and the deepening clinical translation of NGS-based diagnostics. Recent advances in high-throughput sequencing have focused on three major directions: ultrafast genomics has reduced whole-genome sequencing turnaround time to under four hours, enabling same-day rapid diagnosis for critically ill patients [120, 121]; long-read sequencing has generated the world's largest dataset of full-length RNA transcripts, facilitating detection of complex isoforms and previously elusive transcripts [122, 123]; and deepening clinical translation has yielded automated NGS systems capable of returning results within 24 h, expanding access to precision oncology in general hospitals [124]. Collectively, these advances are driving high-throughput sequencing from research centers toward routine clinical laboratories, laying a foundation for the widespread implementation of liquid biopsy.

### Antibody-based detection strategies

Although liquid biopsy has been predominantly driven by nucleic acid detection (e.g., ctDNA), antibody-based strategies play an equally critical role, particularly in the detection of circulating tumor cells (CTCs) and protein biomarkers. In the context of liquid biopsy, antibody-based methods address analytes that nucleic acid approaches cannot directly access: intact cells (CTCs) and protein-level biomarkers that reflect tumor biology, immune response, or functional status. These methods are essential because not all tumor-derived information is encoded in DNA—post-translational protein modifications, cell surface marker expression, and host immune responses provide complementary layers of diagnostic information.

For CTC enumeration, the CellSearch system remains the only FDA-approved platform for clinical use. This technology employs anti-EpCAM (epithelial cell adhesion molecule) antibodies for immunomagnetic capture, followed by verification and counting using anti-cytokeratin (CK) antibodies to identify epithelial-derived tumor cells and anti-CD45 antibodies to exclude leukocytes [125]. In liquid biopsy, CTC enumeration via CellSearch has been clinically validated for prognostic assessment in metastatic breast, colorectal, and prostate cancer, where elevated CTC counts correlate with poorer overall survival and disease progression [15–18]. Beyond enumeration, antibody-based CTC capture enables downstream molecular characterization—including single-cell genomic analysis and organoid culture—providing functional insights that ctDNA alone cannot offer [22–24]. Its primary clinical applications include prognostic assessment and treatment monitoring in metastatic cancers [126]. For protein biomarker detection, several ultra-sensitive methods have been developed beyond conventional ELISA. These include technologies such as Single-Molecule Array (Simoa), Immuno-PCR, Electrochemiluminescence (ECL), and Proximity Extension Assay (PEA). In liquid biopsy, these methods are applied to detect proteins that are shed from tumors or released into circulation at extremely low concentrations—often in the fg/mL to pg/mL range—where conventional ELISA lacks sufficient sensitivity. Simoa is a digital immunoassay that achieves fg/mL-level sensitivity by isolating immune complexes in femtoliter-sized microwells and counting individual fluorescent signals; it has been successfully applied to detect ultra-low abundance proteins such as neurofilament light chain for neurological disease monitoring and phosphorylated tau species for Alzheimer's disease diagnosis from blood samples [127, 128], and in oncology, it enables detection of circulating tumor proteins at early disease stages where conventional immunoassays fail.

Immuno-PCR, which conjugates antibodies with DNA reporters and amplifies the signal via qPCR, achieves 100–10,000 × higher sensitivity than ELISA and is particularly valuable for detecting low-abundance pathogen antigens during seroconversion windows and for validating rare protein biomarkers in samples where only minute volumes are available [129]. Electrochemiluminescence generates light signals through electrochemical reactions at electrode surfaces, offering high sensitivity and an extremely wide dynamic range (> 6 logs); ECL-based platforms have been widely adopted in clinical settings for routine measurement of established tumor markers (e.g., CEA, PSA, CA125), cardiac troponin, and infectious disease markers due to their high automation, clinical validation, and suitability for high-throughput laboratory environments [130–132]. Proximity Extension Assay uses pairs of antibodies carrying complementary DNA probes that only hybridize and extend when both antibodies bind the same target molecule, providing ultra-high specificity through dual recognition; PEA is particularly suited for unbiased proteomic screening and multiplex biomarker discovery, enabling simultaneous quantification of hundreds of proteins from minute serum volumes—an application critical for identifying novel circulating protein biomarkers from limited patient samples [133, 134].

The selection of these antibody-based techniques in liquid biopsy is based on the particular analytical requirements: Simoa has the maximum sensitivity for the single-plex determination of very low protein concentrations, Immuno-PCR shows great sensitivity but with a complicated procedure, ECL combines sensitivity, dynamic range and automation for common clinical tests, and PEA permits high-plex discovery from limited samples (Table 4).

**Table 4:** Comparison of antibody-based detection methods

Method	Core Principle	Sensitivity (Fold Improvement vs. ELISA)	Key Advantages	Major Limitations	Typical Applications	References
**Simoa** (Single-Molecule Array)	Digital immunoassay: immune complexes are sealed in femtoliter-sized microwells for single-molecule counting	100–1,000 × (fg/mL range)	Highest sensitivity (gold standard); single-molecule detection; absolute quantification; commercially mature platform	High cost; relatively low throughput; expensive instrumentation	Neuroscience (blood-based detection of Aβ, Tau); early cancer screening (ultra-low abundance CTC proteins); cytokine quantification	[127]
**Immuno-PCR**	Antibody conjugated to DNA reporter; signal amplified via qPCR	100–10,000 × (fg/mL range)	Ultra-high sensitivity; leverages existing PCR infrastructure; wide dynamic range	Complex conjugate preparation; prone to DNA contamination; labor-intensive workflow	Detection of low-abundance pathogen antigens during seroconversion window; validation of rare proteins; single-plex assays requiring extreme sensitivity	[129]
**Electrochemiluminescence (ECL)**	Electrochemical reaction at electrode surface generates light signal; reduces background	10–100 × (fg/mL-pg/mL range)	High sensitivity; extremely wide dynamic range (> 6 logs); high automation; clinically validated	Slightly lower sensitivity than Simoa/IPCR; expensive equipment	High-throughput clinical labs (tumor markers CEA/PSA; infectious disease screening; cardiac markers troponin); drug screening	[130–132]
**Proximity Extension Assay (PEA)**	Paired antibodies carry complementary DNA probes; upon binding same target, probes hybridize and extend; detected by qPCR/NGS	100–1,000 × (fg/mL range)	Ultra-high specificity (dual antibody requirement); suitable for ultra-multiplex detection; very low background	Technically complex; requires specialized reagents/instrumentation; high cost	Biomarker discovery (unbiased proteomic screening); signaling pathway analysis (multiplex quantification of phosphorylated proteins); cytokine profiling	[133, 134]

### Flow cytometry

Flow cytometry is applied to a quick and multi-parameter quantitative analysis and sorting of individual cells or small particles dissolved in a liquid. In comparison with the conventional liquid biopsy which is based on circulating free DNA (cfDNA), the combination of flow cytometry can identify both intact circulating tumour cells (CTCs) and extracellular vesicles (EVs), including the variations at 'molecular' and 'cellular' degrees. In the area of liquid biopsy, flow cytometry satisfies the two main objectives which cannot be accomplished by the nucleic acid detection methods: (i) the enumeration and phenotypical investigation of rare intact CTCs in blood, and (ii) the high speed and multi-parameter examination of EV subpopulations without any previous separation.

For CTC detection, cells are typically enriched from blood based on size (e.g., microfiltration membranes) or surface markers (e.g., immunomagnetic beads targeting EpCAM). Subsequently, flow cytometry enables high-sensitivity, high-specificity identification of these extremely rare cells from among billions of blood cells. Isolated CTCs can then be analyzed for genetic mutations, providing tumor-specific information that cfDNA analysis alone may fail to capture [135]. In liquid biopsy, flow cytometry-based CTC analysis has been applied to enumerate CTCs across various cancer types, with elevated CTC counts serving as a prognostic marker for progression-free and overall survival [15–18]. More importantly, flow cytometry enables phenotypic characterization of CTCs—such as epithelial (EpCAM +/CK +), mesenchymal (vimentin +), or hybrid states—providing insights into metastatic potential and treatment response that cannot be inferred from ctDNA alone [123]. For EV analysis, nanoscale flow cytometry (nanoFCM) enables single-particle detection of EVs directly in biofluids such as plasma, circumventing laborious isolation steps. EV-associated proteins (e.g., PSMA) can serve as potential biomarkers for cancer recurrence and treatment resistance monitoring [136]. In liquid biopsy, flow cytometry-based EV analysis offers several unique advantages: (i) it requires minimal sample volume and processing time compared to ultracentrifugation or precipitation-based methods; (ii) it enables multiparameter analysis of EV surface markers simultaneously, allowing discrimination of tumor-derived EVs from those originating from platelets, endothelial cells, or immune cells; and (iii) it can be combined with fluorescent probes to detect EV-associated nucleic acids or proteins in a high-throughput manner [119, 120].

However, several challenges persist. EV analysis often relies on traditional manual gating strategies, which are time-consuming and subjective, highlighting the need for standardized approaches. Additionally, dependence on single markers (e.g., EpCAM) risks false negatives, necessitating multi-marker combinatorial strategies. These challenges are particularly relevant in liquid biopsy, where CTCs and tumor-derived EVs are extremely rare events (as few as 1–10 CTCs per mL of blood), requiring robust gating strategies to distinguish true signals from background noise. To address these limitations, flow cytometry is increasingly integrated with cutting-edge technologies. Automated gating algorithms and artificial intelligence (AI)-driven sorting are being developed to eliminate subjectivity and improve analytical consistency and efficiency [137]. Furthermore, integrating flow cytometry with downstream genomic analysis—such as sequencing or digital PCR of sorted CTCs—or combining cytometric data with cfDNA sequencing and secreted proteomics enables construction of comprehensive tumor atlases [138]. In the liquid biopsy workflow, these integrated approaches enable a "phenotype-first, genotype-second" strategy: flow cytometry first identifies and sorts rare CTCs or EV subpopulations based on surface marker profiles, followed by targeted genomic or transcriptomic analysis of these sorted populations, providing functional and molecular characterization that neither approach alone can achieve.

### Single-cell transcriptome analysis

Single-cell transcriptome analysis (scRNA-seq) can determine all the messenger RNA (mRNA) in every cell, offering a more detailed investigation of cellular heterogeneity. In the area of liquid biopsy, the primary application of scRNA-seq is to study circulating tumour cells (CTCs). Compared with the current ctDNA-based liquid biopsy technique which can only identify the overall level of tumour DNA, scRNA-seq highlights the remarkable heterogeneity in the CTC population [139]. In liquid biopsy, scRNA-seq can overcome one of the main drawbacks of the traditional bulk analysis methods: the incapability to distinguish between various subpopulations of CTCs which may have different metastatic potentials, drug sensitivities or resistance mechanisms. By analysing the transcriptome of each CTC, scRNA-seq can reflect the functional condition of each cell like the activation of certain signalling pathways, epithelial-mesenchymal transition (EMT) state and the expression levels of drug resistance genes, thus providing important knowledge about the tumour biology and being beneficial for precise medicine.

This approach allows identification of CTC subpopulations exhibiting epithelial, mesenchymal, or intermediate epithelial-mesenchymal transition (EMT) phenotypes, providing direct insights into metastatic potential. For instance, studies have demonstrated that CTC subsets with mesenchymal or stem-like characteristics are associated with poorer prognosis [140]. In clinical liquid biopsy, this phenotypic information provides direct insights into metastatic potential and disease progression [140, 141]. Furthermore, longitudinal scRNA-seq analysis of CTCs collected at different time points (e.g., pre-treatment, during therapy, at recurrence) or comparison with metastatic lesions can identify "metastasis-initiating cells" with high metastatic potential, treatment resistance, or dormancy capabilities. Characterizing the gene expression signatures of these cells offers critical targets for developing anti-metastatic therapies [141]. In the liquid biopsy workflow, serial scRNA-seq of CTCs enables real-time tracking of clonal evolution and the emergence of drug-resistant populations in response to therapeutic pressure [142].

Beyond CTCs, scRNA-seq can be applied to circulating immune cells in peripheral blood. Tumor-reactive T cells and myeloid-derived suppressor cells, in particular, reflect the immunosuppressive or activated state of the tumor microenvironment [143]. Non-invasive scRNA-seq profiling of peripheral immune cells enables dynamic monitoring of systemic immune responses to immunotherapies such as immune checkpoint inhibitors, facilitating early prediction of treatment efficacy and identification of resistance mechanisms [144]. In liquid biopsy, this application is particularly valuable because it provides a window into the tumor-immune interaction without requiring invasive tumor tissue sampling. Changes in the transcriptomic profiles of circulating T cells, natural killer cells, or monocytes can serve as early indicators of response or resistance to immunotherapy.

Remarkably, serial scRNA-seq analysis of CTCs throughout treatment enables real-time tracking of tumor cell adaptive responses to therapeutic stress, revealing non-genetic mechanisms of drug resistance and informing combination therapy strategies [142]. This capability allows scRNA-seq to capture non-genetic mechanisms of drug resistance (e.g., transient upregulation of survival pathways or drug-tolerant persister states) that are not detectable through ctDNA mutation analysis alone, highlighting its complementary value for comprehensive resistance monitoring.

Despite its promise, clinical application of scRNA-seq in liquid biopsy faces substantial challenges. CTCs are extremely rare in blood and are susceptible to stress-induced damage or apoptosis during isolation. Efficient capture and acquisition of high-quality whole-transcriptome data from these rare cells at scale remains a technical bottleneck [145, 146]. In the liquid biopsy context, these challenges are magnified because the rarity of CTCs (often 1–10 cells per mL of blood) demands ultra-sensitive capture methods and low-input library preparation protocols. Furthermore, the stress of isolation and sorting can alter gene expression profiles, potentially introducing artifacts that complicate data interpretation. Future directions include integrating CTC transcriptomic profiles with spatial information from primary or metastatic tumors to infer CTC tissue of origin and shedding mechanisms. Simultaneous multi-omic analysis of the same CTC—integrating genomic (mutations), transcriptomic (expression), and epigenomic data—will provide a comprehensive picture linking driver mutations to functional states. Ultimately, translating these findings into clinically useful biomarkers will require large-scale prospective studies and establishment of standardized protocols for routine clinical implementation [146]. From a liquid biopsy perspective, the integration of scRNA-seq with other modalities—such as ctDNA sequencing for mutation tracking and spatial transcriptomics for tissue context—represents a promising direction for constructing a complete, multi-dimensional view of tumor evolution and dissemination.

### Biosensors and electrochemical detection

Biosensors are analytical devices that convert biological recognition events into measurable signals. Their core functionality relies on the synergy between highly specific biological recognition elements and highly sensitive signal transducers [147]. In liquid biopsy applications, recognition elements—such as aptamers, antibodies, or nucleic acid probes—are designed to specifically capture target biomarkers, including mutation-specific ctDNA fragments, CTC surface antigens, or tumor-derived proteins in blood. Electrochemical detection serves as a particularly effective "signal translator" in this context. Upon target binding to the recognition element, subtle changes occur in the electrochemical properties of the electrode interface (e.g., current, voltage, or impedance). These changes can be precisely quantified, enabling ultra-sensitive detection of the target analyte. In the context of liquid biopsy, biosensors and electrochemical detection address a critical unmet need: bringing liquid biopsy analysis from centralized laboratories to point-of-care settings. Unlike PCR or NGS-based methods that require sophisticated instrumentation, thermal cycling, or extensive data processing, electrochemical biosensors can deliver rapid (minutes), low-cost, and portable detection—capabilities that are essential for implementing liquid biopsy in primary care, resource-limited settings, or even home-based monitoring [148].

This method has great feasibility in many fields. For instance, electrochemical biosensors can count CTCs without labeling and directly, providing real-time information for cancer diagnosis [149]. The systems that combine the CRISPR-Cas system with electrochemical measurements can detect low-frequency ctDNA mutations at a low cost and quickly, which may provide possibilities for early cancer warnings and companion diagnostics for targeted therapies [150].

Some applications of electrochemical biosensors in liquid biopsies, such as the on-site detection of CTCs, use biosensors coated with antibodies or aptamers specific to EpCAM or other CTC surface markers to capture and quantify CTCs from blood samples with little processing in a few minutes, without the need for immunomagnetic separation or flow cytometry [148, 149]. By combining the CRISPR-Cas system with electrochemical measurements, researchers have also established platforms able to detect low-abundant ctDNA mutations like EGFR T790M and KRAS G12D with high sensitivity and specificity, serving as an economic and fast substitute for ddPCR or NGS for observing known mutations [150]. Furthermore, new multi-channel electrochemical sensors can determine multiple targets simultaneously, such as different ctDNA mutations or combinations of ctDNA and protein biomarkers, in one experiment, solving the multiplexing problem of conventional electrochemical methods [151]. Besides, the sample preparation and electrochemical detection can be integrated into microfluidic "lab-on-a-chip" devices, which can perform the "sample-to-answer" operation with less user interference, an important feature for clinical application [152].

Compared to conventional next-generation sequencing or digital PCR, electrochemical biosensors have advantages in detection speed (minutes instead of hours), portability (portable devices) and per-test cost, which may allow the application of liquid biopsy in primary cases and even at home monitoring [148]. In the liquid biopsy procedure, electrochemical biosensors are suitable for screening or monitoring the known targets rather than discovery tools. For instance, they can be used for the regular monitoring of a known specific mutation during targeted therapy or for rapid screening in primary health care. If a positive signal is detected, the patients can be referred to confirm their condition by performing a comprehensive profile based on next generation sequencing. This will establish a diagnostic plan taking into consideration the aspects of cost, speed and comprehensiveness. Future researches are striving to enhance the liquid biopsy technique with higher multiplexing, easy integration and the utilization of intelligent systems. Some new trends involve the creation of multi-channel electrochemical sensors which can detect multiple markers at the same time [151]; the combination of sample processing and detection in microfluidic "lab-on-a-chip" systems to achieve a continuous operation from "sample-to-answer" [152]; and the application of artificial intelligence algorithms to extract diagnostic information from electrochemical signals [153]. In summary, biosensors and electrochemical detection not only provide technical tools for achieving highly sensitive liquid biopsy analysis but also represent a powerful force driving the translation of liquid biopsy from specialized laboratories into routine clinical practice, ultimately advancing the goal of universally accessible precision medicine.

### Conclusion of detection methods

**Fig. 3:**
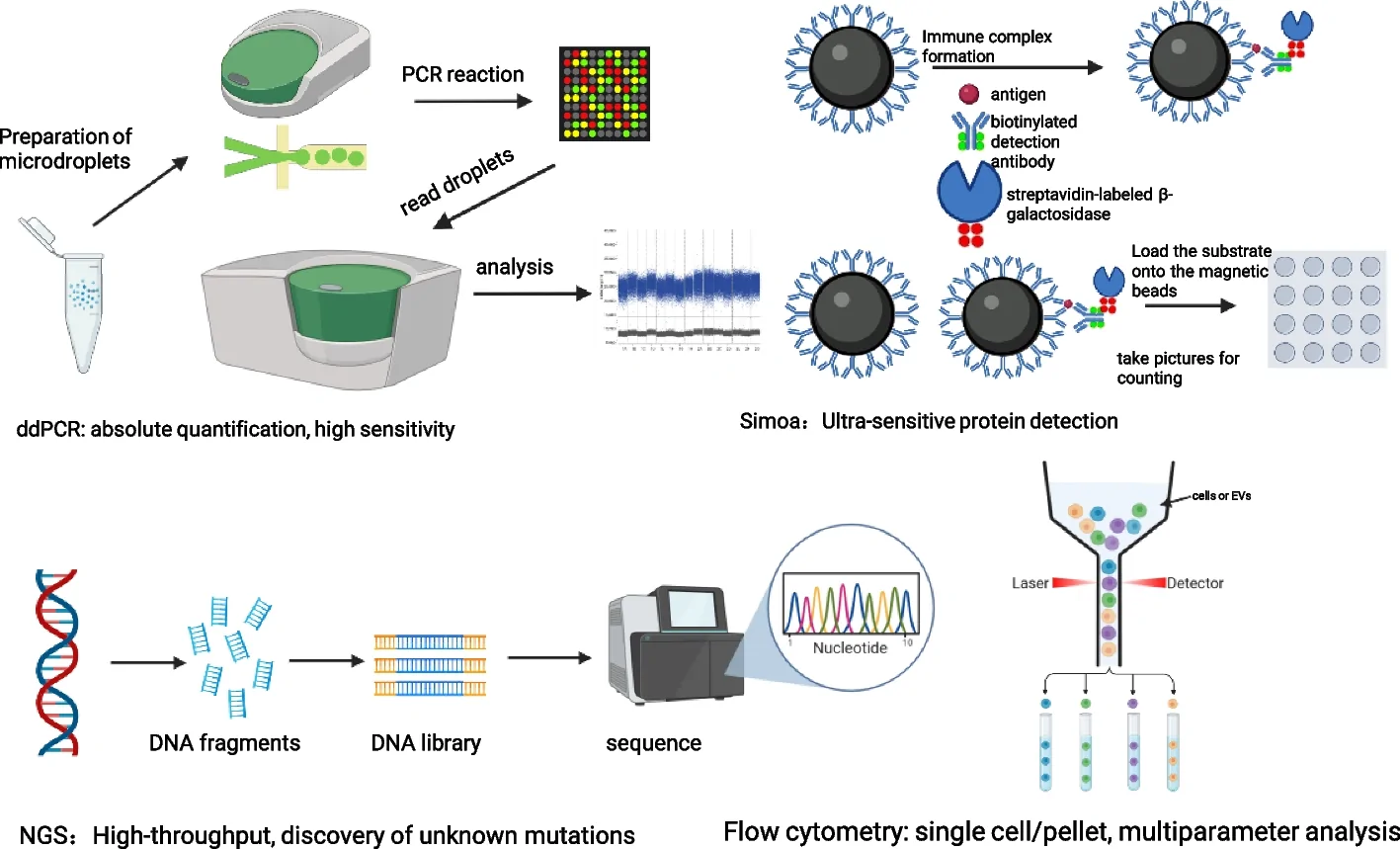
Comparison of core detection technologies in liquid biopsy. This schematic contrasts the core workflows of ddPCR, next-generation sequencing (NGS), the Single Molecule Array (Simoa), and flow cytometry, illustrating how each platform achieves absolute quantification of nucleic acids, discovery of unknown mutations, ultra-sensitive protein detection, and multi-parameter analysis of single cells/particles, respectively

In summary, the liquid biopsy detection method has many applications and is developing quickly with the increasing requirement for high sensitivity and specificity. PCR-based methods, especially ddPCR, can accurately measure the amount of known mutations by sample separation, which is appropriate for assessing the therapeutic effects and diagnosing residual diseases. However, next generation sequencing (NGS) techniques provide a fast and unbiased platform for detecting unknown mutations and analyzing the whole genetic characteristics, being important for studying the heterogeneity of tumors and exploring the drug resistance mechanisms. Besides the nucleic acid tests, antibody-based techniques and flow cytometry can also detect sensitive proteins and analyze the cells of CTCs and EVs. Moreover, some new areas like single cell transcriptomics and electrochemical biosensors are expected to give more detailed molecular information and point of care applications. To explain the main steps of these major methods, a comparative schematic has been shown in Fig. 3, illustrating the basic procedures of ddPCR, next-generation sequencing (NGS), Single Molecule Array (Simoa) and flow cytometry, demonstrating how each system obtains different kinds of molecular information from the blood samples. The combined use and intelligent application of these various tools will be beneficial for the implementation of liquid biopsy in daily medical treatment.

## Clinical application and translation of liquid biopsy in Oncology

### Early cancer detection

Early detection remains the most critical factor in reducing cancer-related mortality. Traditional screening modalities—including various imaging techniques and endoscopic examinations—have proven effective for certain cancer types but are limited by invasiveness, radiation exposure, and variable sensitivity. Liquid biopsy, particularly ctDNA-based assays, offers the promise of non-invasive, scalable early cancer screening that could complement or, in some cases, replace existing approaches [107].

The central challenge for clinical translation of liquid biopsy in early detection is twofold. First, the concentration of ctDNA in blood from patients with early-stage disease is extremely low, typically < 0.1% mutant allele frequency, demanding ultra-high technical sensitivity. Second, and more fundamentally, biological noise—including mutations arising from clonal hematopoiesis of indeterminate potential (CHIP), cfDNA shed from normal tissues, and inter-tumor heterogeneity—can confound signal interpretation even when technical sensitivity is adequate [154]. Therefore, the challenge is not only reaching 0.001% sensitivity but also distinguishing true tumor-derived signals from biological background at that level. Currently, four primary strategies are being explored to address this challenge: ctDNA mutation analysis, ctDNA methylation profiling, multi-cancer early detection (MCED) approaches, and protein-based biomarkers. ctDNA mutation analysis using targeted NGS directly identifies actionable driver mutations but has limited sensitivity in early-stage patients due to low ctDNA shedding [155, 156]. ctDNA methylation analysis offers high accuracy in tissue-of-origin localization, though it requires extensive reference methylation databases [111]. MCED assays integrate mutations, methylation, and multi-omics data analyzed by machine learning to screen multiple cancer types simultaneously, yet face challenges in managing false positives [112]. Protein biomarkers, detected by antibody microarrays, ELISA, or Simoa, are cost-effective and easily implemented but require composite panels to achieve adequate sensitivity and specificity [163–165].

#### Methylation-based markers

ctDNA methylation patterns exhibit high tissue specificity and cancer-type specificity, with alterations often detectable even in early-stage disease. By characterizing the methylation profile of ctDNA in peripheral blood, it is possible not only to determine whether an individual has cancer but also to predict the tissue of origin with high accuracy—a critical capability for guiding subsequent clinical workup [157]. Compared to somatic mutation-based detection, methylation analysis generally demonstrates superior sensitivity in early-stage cancers, as methylation changes are more abundant and occur earlier in tumorigenesis [157]. For instance, a study by Chen et al. demonstrated that ctDNA methylation-based analysis could detect liver cancer up to four years before conventional diagnosis, with a specificity of 95% [12]. Similarly, a multimodal ctDNA-based assay named SPOT-MAS, which integrates methylation and fragmentomic profiles, achieved a sensitivity of 96.8% at 97% specificity in detecting nonmetastatic colorectal cancer, demonstrating the high accuracy of methylation-based liquid biopsy for early-stage disease detection [158].

#### Multi-cancer early detection (MCED)

MCED assays aim to screen for multiple cancer types simultaneously from a single blood draw. These tests detect common biomarkers shared across multiple cancers, such as combinations of mutations and methylation signatures. Landmark studies, most notably the Circulating Cell-free Genome Atlas (CCGA) study by GRAIL, have demonstrated that analyzing multidimensional ctDNA features using large-scale machine learning models can enable detection of dozens of cancer types in presymptomatic populations while achieving high accuracy in tissue-of-origin localization [111, 112]. These advances represent a paradigm shift from single-cancer screening to comprehensive, multi-cancer early detection strategies. The Galleri test, developed by GRAIL, is a commercially available MCED assay that screens for more than 50 cancer types from a single blood draw by detecting cancer-specific methylation patterns in circulating cell-free DNA. In the prospective PATHFINDER study involving 6,621 participants without cancer symptoms, the test demonstrated a positive predictive value of 38.0%, and among participants with a confirmed cancer diagnosis, 71% had early-stage (I-III) disease, including cancers without established screening guidelines such as liver and pancreatic cancers [159].

Despite its promising prospects, liquid biopsy for early cancer screening still faces many translational challenges. Specificity must be extremely high (> 99%) to avoid a large number of false-positive results, which could lead to unnecessary invasive procedures and patient anxiety. Cost-effectiveness also needs to be validated in large population-based studies. In addition, the clinical management and intervention for individuals who screen positive but with negative imaging findings remain a challenge that has not been fully addressed. It is also critical to distinguish between technical sensitivity and clinical sensitivity. While advanced technologies such as ddPCR and NGS can theoretically detect mutations at 0.001% mutant allele frequency under ideal conditions, the practical limit for reliable early cancer detection is dictated by biological noise—including clonal hematopoiesis (CHIP)-related mutations, cfDNA shed from normal tissues, and inter-tumor heterogeneity—rather than technical constraints alone. These biological factors impose a practical detection floor typically around 0.1% MAF for clinically actionable decisions, explaining why the < 0.1% MAF observed in early-stage patients remains a genuine challenge despite the availability of ultra-sensitive detection platforms.

In the future, with improvements in sequencing technology, optimization of bioinformatics algorithms and large-scale prospective clinical trials, the outcomes of liquid biopsy are anticipated to be incorporated into the regular cancer screening procedure. This will facilitate the final objective of early detection, early diagnosis and early treatment, significantly enhancing the patient's prognosis.

### Predict response to chemotherapy, radiotherapy, immunotherapy and primary drug resistance

Traditionally, treatment options for tumors have relied heavily on population-based clinical trial data and limited biomarkers. Liquid biopsy can reveal the genomic characteristics of tumors in near real time, so that the response can be predicted before the initiation of treatment or early, and the ineffective treatment and its toxic side effects can be avoided.

#### Prediction of chemotherapy response

ctDNA analysis can be used to evaluate the genetic state associated with chemotherapy sensitivity. For example, in colorectal cancer, detecting RAS mutations in ctDNA is a key indicator of resistance to EGFR-targeted therapies (often used in combination with chemotherapy). If RAS mutations are detected, it suggests primary resistance to drugs such as cetuximab [160]. In addition, certain genetic signatures (e.g., high ERCC1 expression associated with platinum resistance) can be indirectly assessed through ctDNA methylation or fragmentation analysis, providing a basis for personalized chemotherapy selection [161]. For example, a prospective study of 137 patients with metastatic colorectal cancer receiving cetuximab-based therapy found that those with detectable RAS mutations in ctDNA at baseline had a median progression-free survival of only 2.1 months compared to 9.4 months in RAS wild-type patients (hazard ratio 4.2, 95% CI 2.7–6.5), demonstrating the strong negative predictive value of ctDNA RAS testing [160].

#### Prediction of radiotherapy response

Although still in early stages, liquid biopsy holds promise in radiotherapy. In theory, radiation kills tumor cells and releases ctDNA. By monitoring the dynamic changes (clearance rate) of ctDNA before and after radiotherapy, tumor sensitivity to radiation can be assessed early. A rapid decrease in ctDNA levels post-radiotherapy often indicates a good therapeutic response, whereas stable or rising levels may suggest primary resistance [162]. Furthermore, using liquid biopsy to identify radioresistance-associated mutations (e.g., TP53, KRAS) can help identify patients unlikely to benefit from treatment beforehand, allowing for treatment plan adjustments [163, 164]. In a study of patients with locally advanced head and neck cancer undergoing chemoradiotherapy, those who achieved a > 50% reduction in ctDNA levels by the second week of treatment had significantly higher 2-year progression-free survival (78% vs. 32%, p < 0.01) compared to those with persistent ctDNA [163].

#### Prediction of immunotherapy response

This is one of the most active areas of liquid biopsy application. The efficacy of immune checkpoint inhibitors (ICIs) is highly dependent on the tumor microenvironment, and liquid biopsy provides a unique perspective [165]. Blood-based tumor mutational burden (bTMB) can be calculated via ctDNA sequencing. High bTMB typically indicates more neoantigen production and has been validated in multiple studies as a predictor of better response to ICIs [108, 109]. In addition, detection of MSI status (bMSI) via ctDNA has emerged as a rapid, non-invasive method for early identification of colon cancer patients likely to benefit from ICIs [110]. Early ctDNA kinetics—defined as a sharp decrease in ctDNA levels within 2–4 weeks of treatment initiation—has proven to be a strong early indicator of long-term immunotherapy efficacy, sometimes preceding radiographic changes [166]. Conversely, stable or increasing ctDNA levels are strongly suggestive of primary resistance [167]. In a phase II trial of atezolizumab in NSCLC, patients with bTMB ≥ 16 mutations per megabase had significantly longer progression-free survival (median 6.3 vs. 2.9 months, HR 0.60, p = 0.008) compared to those with bTMB < 16 [109]. For MSI status, a prospective study of 107 colorectal cancer patients receiving pembrolizumab reported that bMSI-positive patients achieved an objective response rate of 58%, compared to only 5% in bMSI-negative patients [110].

In conclusion, by integrating multiple data types such as gene mutations, methylation, and bTMB, liquid biopsy can construct comprehensive predictive models to identify patients likely to exhibit primary resistance to chemotherapy, radiotherapy, or immunotherapy before treatment begins. This lays the foundation for true "precision oncology," enabling doctors to avoid ineffective regimens and turn to alternative strategies or clinical trials, thereby maximizing treatment benefits and improving patients' quality of life [168]. Future directions include conducting more prospective clinical trials to confirm the clinical utility of these models and integrate them into standard care guidelines [107].

### Monitoring treatment response and acquired resistance

Traditional efficacy evaluation mainly relies on imaging (e.g., RECIST criteria), but this method has a lag time and cannot distinguish true progression from pseudoprogression [169]. Liquid biopsy can assess treatment response earlier and more accurately by quantitatively analyzing changes in ctDNA levels.

During chemotherapy, targeted therapy, or immunotherapy, a rapid decline in ctDNA levels is significantly associated with good treatment response and longer survival [167]. In addition, liquid biopsy is one of the most sensitive tools for detecting minimal residual disease (MRD) after radical surgery or chemoradiotherapy. By using individualized sequencing methods or ultra-high sensitivity panels to regularly monitor patients' blood, it is possible to detect molecular signs of relapse months or even years prior to imaging-confirmed recurrence [11]. MRD-positive status is a powerful predictor of clinical relapse risk, opening new avenues for identifying high-risk patients who require adjuvant therapy and for determining treatment duration. For example, in a landmark study of patients with localized lung cancer who underwent curative-intent therapy, detection of ctDNA after treatment predicted radiographic relapse with 100% sensitivity and 90% specificity, with a median lead time of 5.2 months before clinical or radiographic progression [11]. This early warning window allows timely intervention, such as initiating adjuvant therapy before macroscopic disease becomes detectable.

A major challenge of targeted therapies is the almost inevitable development of acquired resistance. When tumors progress, repeat tissue biopsies are often difficult and risky. Liquid biopsy offers a safe and reproducible alternative, comprehensively capturing new resistant clones arising from tumors under therapeutic pressure. For example, in the AURA3 trial of patients with EGFR-mutant NSCLC who developed acquired resistance to first-generation EGFR-TKIs, plasma genotyping using ddPCR detected the T790M resistance mutation in 44% of patients. The concordance between plasma and tissue-based T790M detection was 87%, and importantly, patients with T790M detected in plasma derived similar clinical benefit from osimertinib (median progression-free survival 10.1 months) as those identified by tissue biopsy (9.5 months), validating the clinical utility of liquid biopsy for resistance monitoring [99]. Similarly, in anti-EGFR therapy for colorectal cancer, liquid biopsy can detect KRAS/NRAS mutations that emerge during treatment, clearly revealing the cause of resistance [170, 171]. In addition to point mutations, liquid biopsy can identify gene amplifications (e.g., MET amplification) and fusions (e.g., ALK fusion) associated with drug resistance, providing a comprehensive molecular map for overcoming resistance [107, 113, 172].

Liquid biopsy is becoming a clinical method for evaluating the treatment effect and detecting drug resistance. It brings about a change from a "fixed" diagnosis to a "changing" observation, allowing the cancer treatment to be an individualized management which can be modified in real time [173]. The future developments involve confirming the use of ctDNA monitoring as a substitute endpoint in clinical trials and carrying out clinical studies with interventions based on MRD and resistance mutation test results, thus achieving long-term and accurate control of cancer [174].

### Analysis of tumor heterogeneity for treatment selection and precision medicine

Tumor heterogeneity, including spatial heterogeneity and temporal heterogeneity, is the core challenge leading to treatment failure and disease progression [175]. Traditional biopsy of a single site can only provide an "instantaneous snapshot" of the tumor genome, which cannot fully reflect the whole picture of the tumor. Liquid biopsy provides a comprehensive and integrated "molecular panorama" by capturing circulating tumor DNA (ctDNA) released by tumor lesions from different locations at different times, which plays a revolutionary role in revealing tumor heterogeneity, guiding treatment selection and promoting precision medicine.

#### Overcoming spatial heterogeneity

There may be significant genetic differences within a tumor (intratumor heterogeneity) or between the primary and metastatic sites (intertumoral heterogeneity) [175]. A single tissue biopsy may miss driver mutations in some subclones, leading to incomplete treatment selection or misinterpretation of the risk of resistance. Liquid biopsy is theoretically capable of capturing ctDNA released from all active lesions, thereby providing a "comprehensive genetic report" that represents the overall tumor burden [107]. For example, in advanced lung cancer, ctDNA can often detect drug-resistance mutations such as T790M that are negative on tissue biopsy, indicating the direction for subsequent therapy. For example, in advanced breast cancer or lung cancer, liquid biopsy may simultaneously detect specific drug-resistance mutations or targetable genetic alterations that are not found on tissue biopsy, allowing treatment strategies to reach a broader tumor cell population. Thus, early treatment failure caused by "blind spots" can be avoided.

#### Temporal heterogeneity of dynamic monitoring:

Tumors will undergo clonal evolution under treatment selection and drug pressure, which is the main mechanism of acquired drug resistance [176]. The non-invasive and reproducible properties of liquid biopsy make it an ideal tool to track the dynamic changes of tumor genomes over time. By analyzing serial samples, physicians can delineate the complete process by which sensitive clones are eliminated and resistant clones are gradually enriched. This dynamic perspective is essential for precision medicine: it can not only explain "why the current treatment is not working," but also predict "what treatment is likely to work next [177]. For example, in a landmark study of 24 colorectal cancer patients receiving anti-EGFR therapy, longitudinal ctDNA analysis revealed that KRAS-mutant clones emerged as early as 5–10 months before radiographic disease progression in 12 of 24 patients (50%). Critically, when anti-EGFR therapy was withdrawn, these mutant clones became undetectable in the majority of patients, demonstrating the reversibility of acquired resistance at the molecular level and providing a rational basis for treatment rechallenge strategies [178].

#### Identifying co-occurring mutations and guiding combination therapy:

The complexity of tumors often results from the interaction of multiple genetic alterations [179]. The comprehensive genomic map provided by liquid biopsy can help identify co-occurring driver mutations, and this information can be important in guiding the selection of the most effective treatment, especially combination strategies [107]. For example, in non-small-cell lung cancer, the simultaneous detection of EGFR-sensitive mutations and MET amplification can explain resistance to first-generation EGFR Tkis and guide precision treatment strategies that combine EGFR Tkis and MET inhibitors [113]. This kind of complex genetic background analysis, based on the liquid biopsy is the key to precision medical higher level.

### Conclusion of clinical applications in oncology

**Fig. 4:**
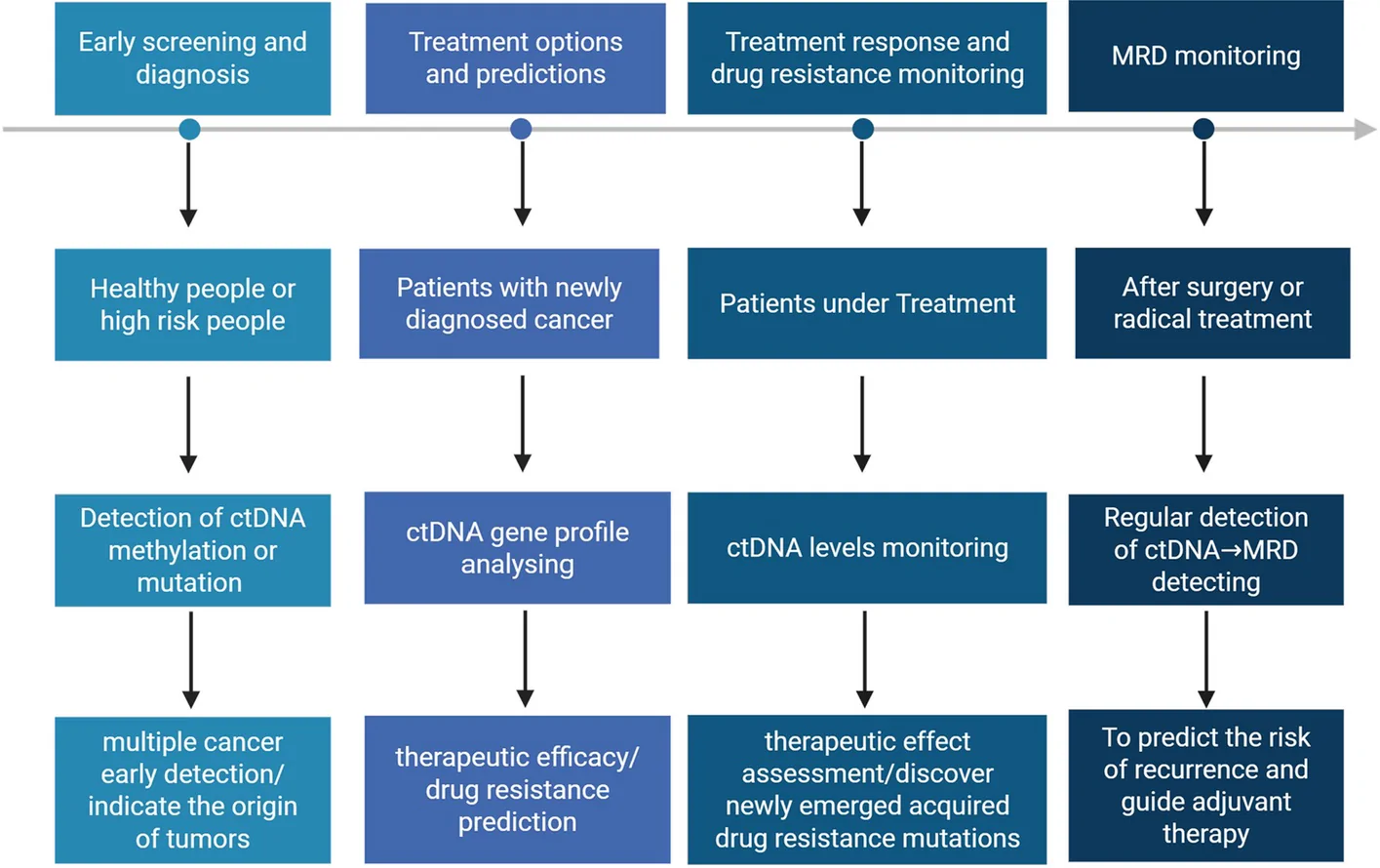
Clinical applications of liquid biopsy across the cancer care continuum. This schematic illustrates the pivotal role of liquid biopsy, primarily through circulating tumor DNA (ctDNA) analysis, at each stage of cancer management. From left to right, the figure demonstrates its utility in: Early screening and diagnosis, where ctDNA methylation or mutation profiling enables multi-cancer early detection (MCED) and prediction of tumor origin; Treatment options and predictions, where genomic analysis guides therapy selection and forecasts potential drug resistance; Treatment response and drug resistance monitoring, where dynamic changes in ctDNA levels facilitate real-time efficacy assessment and early identification of newly acquired resistance mutations; and Minimal residual disease (MRD) monitoring, where regular post-treatment ctDNA surveillance predicts recurrence risk and informs adjuvant therapy decisions for patients following curative-intent surgery or radical treatment

In summary, liquid biopsy has become an important technique in oncology, changing the traditional diagnosis method from tissue based to a personalized medicine based on blood. It has extensive applications throughout the entire cancer treatment process. During the stage of early detection, techniques such as methylation analysis and multi-cancer early detection (MCED) tests provide non-invasive screening with high accuracy of tissues. For the selection of treatment, liquid biopsy can predict the sensitivity to chemotherapy, radiotherapy and immunotherapy by detecting genomic patterns and blood markers like bTMB. Furthermore, its capability to observe the treatment effects through the variation of ctDNA and to discover the new mechanisms of acquired resistance, for example, the EGFR T790M mutation, gives a real-time molecular guide for clinical decisions. Besides, by analyzing the whole genomic structure of complicated tumors, liquid biopsy overcomes the disadvantages of single-point tissue biopsies and directs the rational combination therapies. To demonstrate the significant role of this method during the course of the disease, Fig. 4 illustrates the use of liquid biopsy throughout the entire cancer therapy process, from early screening and treatment selection to the monitoring of response and detection of minimal residual disease (MRD). As large-scale prospective studies are being carried out to confirm its clinical value, liquid biopsy is anticipated to become an indispensable part of the routine oncological treatment, thus improving the treatment results by providing personalized and timely therapies.

## Application of liquid biopsy in non-cancerous diseases

Liquid biopsy technology, with its unique advantages of non-invasiveness and dynamic reflection of the physiological and pathological states of the body, has rapidly expanded from oncology to a broad range of non-cancer diseases. The analytes—circulating cell-free DNA (cfDNA) and extracellular RNA (exRNA), among others—serve as informational messengers distinct from cellular components, providing an unprecedented window into biological processes that are difficult to capture with traditional invasive procedures. At present, this technology is advancing with unprecedented depth and breadth, reshaping the way we diagnose, monitor, and manage a variety of complex diseases. Its core applications at least include the following four key directions: in reproductive medicine, it analyzes fetal genetic material in maternal blood, enabling noninvasive prenatal screening and monitoring of pregnancy complications; in transplantation medicine, detection of donor-derived cfDNA (dd-cfDNA) provides a highly sensitive tool for early warning of allograft injury; in neurological diseases, it has introduced breakthrough blood-based biomarkers for early diagnosis and classification of conditions such as Alzheimer's disease; in infectious diseases, cfDNA-based detection of pathogenic microorganisms has opened a new avenue for rapid pathogen identification in critical infections such as sepsis. This article systematically reviews the key progress and future directions of liquid biopsy in current clinical practice and translational research across these four domains.

### Reproductive medicine

In reproductive medicine, the application of liquid biopsy primarily focuses on specific biomarkers in maternal blood during pregnancy. Maternal peripheral blood contains cell-free fetal DNA (cffDNA), accounting for approximately 10–15% of total maternal cfDNA, providing a unique window for noninvasive access to fetal genetic information [180]. The application of liquid biopsy in reproductive medicine mainly includes four aspects: non-invasive prenatal testing (NIPT), prediction of preeclampsia, non-invasive diagnosis of hereditary monogenic disorders, and applications in assisted reproductive technology.

#### Non-invasive prenatal testing (NIPT)

NIPT is the most mature and widely used successful example of liquid biopsy in the non-oncology field. By analyzing cell-free fetal DNA in maternal plasma through high-throughput sequencing, it can screen for common fetal chromosomal aneuploidies with high accuracy [181]. A large body of evidence has shown that NIPT offers significantly higher sensitivity and specificity compared with traditional serological screening, thereby greatly reducing unnecessary invasive prenatal diagnostic procedures and their associated risk of miscarriage [182]. This advantage has led to its recommendation by mainstream clinical guidelines as a first-line screening option for all pregnant women [183]. Currently, the application of NIPT has extended beyond the initial three types of autosomal aneuploidies to include screening for sex chromosome aneuploidies and certain chromosomal microdeletion/microduplication syndromes, driving continued progress in the field of prenatal screening.

#### Prediction of preeclampsia

Liquid biopsy has been successfully extended to the early prediction of preeclampsia, a serious pregnancy complication. Its pathological basis is placental dysfunction, which leaves molecular 'traces' in maternal plasma cfRNA. Recent breakthrough studies have shown that by analyzing transcriptomic changes in maternal plasma cfRNA weeks or even months before the onset of clinical symptoms, pregnant women at high risk of preeclampsia can be effectively identified [184]. Moreover, cfRNA-based machine learning models have demonstrated excellent predictive performance [184]. This non-invasive prediction method provides a valuable 'lead' for the clinic, enabling early intervention (such as low-dose aspirin prophylaxis) and targeted close monitoring for high-risk pregnant women, which is expected to ultimately improve maternal and infant outcomes [185].

#### Noninvasive diagnosis of hereditary monogenic disorders

For couples at risk of familial monogenic disorders (such as thalassemia, cystic fibrosis, achondroplasia, etc.), invasive procedures have traditionally been required to obtain fetal DNA for diagnosis. By combining parental genotyping with advanced targeted enrichment sequencing technologies to analyze cffDNA in maternal plasma, noninvasive prenatal diagnosis has now been enabled for dozens of monogenic disorders without the risks associated with invasive procedures [186].

#### Applications in assisted reproductive technology

During in vitro fertilization-embryo transfer, embryo aneuploidy is a major cause of implantation failure and early miscarriage [187]. Cutting-edge studies have explored "non-invasive" embryo chromosome screening by detecting cfDNA in embryo culture medium, which is expected to further optimize the process and success rate of assisted reproductive technology [188]. However, the origin of cfDNA in embryo culture medium is complex, and its representativeness and accuracy remain to be improved—these are the main obstacles to the clinical application of non-invasive PGT-A (niPGT-A) at present. The amount of cfDNA in culture medium is small and highly fragmented, and whether it can fully represent the chromosomal status of the entire embryo still requires extensive validation [189]. Therefore, niPGT-A remains in the clinical research stage and has not yet been established as an independent diagnostic method; preimplantation genetic testing through embryo biopsy is still required.

### Transplantation medicine and detection of allograft injury

A major challenge after solid organ transplantation is the timely detection and differentiation of the causes of graft injury, particularly distinguishing acute rejection (AR) from other insults such as infection, drug toxicity, or ischemia–reperfusion injury. Traditional monitoring methods rely on non-specific functional indicators like serum creatinine, while biopsy—the diagnostic "gold standard"—is invasive, carrying risks of bleeding, infection, and graft injury, and cannot be performed frequently. The application of liquid biopsy in transplantation medicine ushers in a new era of precise, non-invasive post-transplant management. By detecting donor-derived circulating cell-free DNA (dd-cfDNA), liquid biopsy has provided a revolutionary tool for the early detection and differential diagnosis of allograft injury.

#### dd-cfDNA as a universal marker of graft damage

cfDNA in the blood of a transplant recipient is a mixture of the recipient's own DNA and donor DNA from the transplanted organ. When cellular damage or death occurs in the graft—whether due to rejection, infection, or other causes—fragments of donor-derived DNA are released into the circulation, resulting in an increase in the proportion or absolute concentration of dd-cfDNA in the blood. Thus, an increase in dd-cfDNA levels serves as a highly sensitive "universal signal" of graft damage [190–194].

#### Early warning and differentiation of rejection

Studies have confirmed that dd-cfDNA levels in blood are significantly elevated during acute rejection, particularly antibody-mediated rejection (ABMR). A key advantage is that this increase often precedes the rise in serum creatinine or the onset of clinical symptoms, providing a valuable "window period" for clinical intervention [190, 195, 196]. In addition, combining dd-cfDNA with traditional immunological markers (such as donor-specific antibodies, DSA) can significantly improve diagnostic accuracy for active rejection and help distinguish it from infection or drug toxicity injury [197].

#### Guiding therapy and risk stratification

dd-cfDNA testing can be used to monitor response to anti-rejection therapy. After successful treatment, a decrease in dd-cfDNA levels provides objective, molecular-level evidence of treatment efficacy [192]. dd-cfDNA can serve as an early and convenient warning of graft injury caused by insufficient immunosuppression. At the same time, sustained low levels of dd-cfDNA in patients with stable graft function provide strong evidence of good graft tolerance [196]. This can help identify low-risk patients who may be suitable for immunosuppressive dose reduction, thereby reducing the toxic side effects of long-term medication [196].

### Disorders of the nervous system

Liquid biopsy technology is improving the blood–brain barrier, which has a good prospect in the detection and observation of nervous system diseases. Formerly, molecular information from the central nervous system was mainly acquired by lumbar puncture or imaging. The former is troublesome and the latter can only diagnose the disease at a late stage. liquid biopsy can offer an alternate method of a non-invasive, early 'molecular window' by examining the biomarkers in the blood which come from the brain.

#### Alzheimer’s disease (AD) and other neurodegenerative diseases

Brain-derived biomarkers in blood are a major focus of current research. For AD, researchers are working to detect phosphorylated Tau (p-tau) protein, the β-amyloid (Aβ) 42/40 ratio, and their presence in exosomes [128, 198]. In recent years, highly sensitive technologies such as the Single Molecule Array (Simoa) and immunoprecipitation-mass spectrometry have enabled accurate measurement of p-tau181 and p-tau217 in peripheral blood. Studies have shown that detection of plasma p-tau181 using the Simoa platform can effectively distinguish AD patients from healthy elderly individuals and those with other dementias. Furthermore, plasma p-tau217 detected by immunoprecipitation-mass spectrometry shows even higher specificity, and its levels correlate strongly with cerebrospinal fluid (CSF) measurements and amyloid (Aβ)-PET imaging, demonstrating high diagnostic value and the ability to distinguish AD from other types of dementia [128, 199, 200]. This provides an unprecedented non-invasive tool for early screening, accurate diagnosis of AD, and for evaluating treatment efficacy in clinical trials.

#### Stroke and neurological injury

After an acute stroke, brain cells rapidly die and release their DNA into the bloodstream. The type and extent of damaged brain cells can be determined by detecting the methylation patterns of cell-type-specific cfDNA (e.g., from neurons, astrocytes, or oligodendrocytes) in the blood [201]. This "cell type-specific" damage profile not only helps assess the severity and prognosis of brain injury, but may also in the future be used to distinguish ischemic from hemorrhagic stroke to guide clinical treatment decisions [201].

#### Neuropsychiatric disorders

Although research is still in its early stages, liquid biopsy has also shown potential in psychiatric disorders such as schizophrenia and depression by analyzing cfDNA methylation profiles, which may reflect disease-related epigenetic changes [202–204]. Numerous challenges remain in this field. First, the concentration of brain-derived biomarkers in blood is very low, necessitating high detection sensitivity. Second, due to the significant heterogeneity of psychiatric disorders, large cohort studies are needed to verify biomarker specificity. In addition, confounding factors such as age, sex, and medication use further increase the complexity of data analysis. Future research directions include developing highly sensitive enrichment and detection techniques, establishing integrated multi-omics analysis strategies, and conducting large-scale prospective cohort studies to validate the clinical value of these biomarkers. With technological advances and further research, liquid biopsy is expected to provide an important tool for the precise diagnosis and treatment of neuropsychiatric diseases.

### Infectious diseases

The application of liquid biopsy in infectious diseases is demonstrating unique value. Unlike traditional methods that require sampling from specific infection sites (e.g., sputum, tissue biopsy), liquid biopsy provides a systemic perspective for the diagnosis and management of infectious diseases by detecting pathogen-derived nucleic acids in the blood. Specifically, it achieves noninvasive diagnosis by analyzing microbial cell-free DNA (mcfDNA) in plasma.

#### Rapid etiological diagnosis of sepsis and bloodstream infections

Traditional blood culture methods require 24–72 h and have a limited positive rate. Metagenomic next-generation sequencing (mNGS) based on plasma cfDNA can identify microorganisms or viruses in patient samples without requiring a priori specification of suspected pathogens, and can complete the entire process from sample collection to result reporting within 24 h, significantly improving diagnostic efficiency [205–208]. This rapid and comprehensive etiological information provides a crucial basis for clinical decision-making, enabling early and accurate anti-infective therapy for critically ill patients.

#### Rapid response to emerging infectious diseases

During outbreaks of emerging infectious diseases, liquid biopsy demonstrates distinct advantages. Traditional reverse transcriptase PCR (RT-PCR) methods for detecting potential carriers are labor-intensive and time-consuming, limiting screening speed in the early stages of an epidemic. Microfluidic technologies, integrated into liquid biopsy workflows, are expected to become promising screening tools [209]. During the COVID-19 pandemic, studies confirmed that detection of SARS-CoV-2 RNA in plasma correlates with disease severity, providing valuable insights for the management of severely ill patients [210, 211].

The application of liquid biopsy in the daily medical care of infectious diseases is now constrained by three main difficulties: the technical challenge of the large amount of human DNA interfering as a background, the financial restriction of the high cost, and the clinical requirement for uniform rules to interpret the results. To overcome these issues, a complete research program is required. This should include enhancing the specific enrichment methods to decrease the background noise, simplifying the automatic analysing procedures for a faster process, and gathering dependable clinical data on the feasibility of quantitative mcfDNA kinetics for assessing the efficacy of the treatment. Ultimately, strict health economic evaluations will be necessary to demonstrate its advantages and promote its use in the regular diagnostic procedures.

Representative applications of liquid biopsy across non-cancerous disease areas—including reproductive medicine, transplantation medicine, neurological disorders, and infectious diseases(Table 5), which highlights the core biomarkers, detection methods, and clinical value for each condition.

**Table 5:** Representative applications of liquid biopsy in the field of noncancer

Disease Areas	Core Biomarkers	Detection Methods	Clinical Application Value	References
Reproductive Medicine	Cell-free fetal DNA (cffDNA)	Massively parallel sequencing (NGS)	Noninvasive, high-precision screening for fetal aneuploidies (e.g., Down syndrome); prediction of preeclampsia; embryo chromosome screening	[181, 184, 186]
Transplantation Medicine	Donor-derived cfDNA (dd-cfDNA)	ddPCR, NGS	Noninvasive, highly sensitive monitoring of graft rejection, with detection preceding functional abnormalities	[190–196]
Neurological Disorders	Plasma p-tau181, p-tau217; Aβ42/40 ratio; cfDNA methylation patterns	Simoa, Immunoprecipitation-mass spectrometry	Noninvasive aid in diagnosis; differential diagnosis of dementia subtypes; monitoring of treatment response	[128, 198–200, 202–204]
Infectious Diseases	Plasma microbial cfDNA (mcfDNA)	Metagenomic sequencing (mNGS)	Rapid (< 24 h), culture-free, and unbiased pathogen identification to guide precise anti-infective therapy	[205–211]

### Application of liquid biopsy in chronic diseases

#### Application in cardiovascular diseases

In the field of cardiovascular diseases, liquid biopsy technology is showing promise for risk stratification and early warning. A pilot study published in 2025 explored the feasibility of integrating whole-blood transcriptomes with artificial intelligence algorithms to predict coronary artery calcification. The prediction model developed in this study combined transcriptome data with clinical risk factors (such as age, sex, and smoking history) and demonstrated high accuracy in identifying coronary calcification (AUC of 0.92), although its upfront cost was still higher than that of traditional coronary calcification screening methods [212]. In addition, circulating cell-free DNA (cfDNA) is gaining attention as a promising biomarker. Studies have found that during myocardial infarction, the cfDNA level rises rapidly within 2 h after chest pain onset, with an elevation time window earlier than that of traditional cardiac troponin, offering the potential for earlier diagnosis and dynamic monitoring [213].

#### Metabolic and fibrotic liver disease

The study of liver diseases, especially metabolic dysfunction-associated steatotic liver disease (MASLD) and its progressive form metabolic dysfunction-associated steatohepatitis (MASH), represents one of the most active areas of liquid biopsy research. MASLD can progress to liver fibrosis, cirrhosis, and even hepatocellular carcinoma, posing a serious threat to human health. Traditional diagnostic methods (such as liver biopsy) are invasive and unsuitable for large-scale screening, while imaging methods have limited sensitivity for early lesions [214]. Liquid biopsy, particularly exRNA-based analysis, offers a noninvasive, reproducible, and highly sensitive alternative. Recent studies have demonstrated that several microRNAs (miRNAs), including miR-122, miR-34a, and miR-21, have been applied in MASLD/MASH research(Table 6).

**Table 6:** Application of exRNA biomarkers in the diagnosis, staging, and monitoring of MASLD

Fields of Application	exRNA Types	Specific Examples	Clinical Relevance/Role	References
**Diagnosis and Staging**	miRNA	miR-122	A core biomarker; positively correlated with hepatic steatosis, inflammation, and fibrosis	[215–220]
	miRNA	miR-34a, miR-21	Associated with disease activity in metabolic dysfunction-associated steatohepatitis (MASH)	[215, 217, 221, 222]
	miRNA panel	miR-192-5p, miR-27b-3p, and others	Can distinguish simple steatosis from MASH when used as a panel	[216, 217, 223–225]
	tsRNA (tRNA-derived fragment)	tRF-Val-CAC-005	Correlates with NAFLD activity score (NAS) and fibrosis stage	[226]
	lncRNA (long non-coding RNA)	NEAT1, MALAT1, HULC	Associated with inflammatory and fibrotic processes in the liver	[227–232]
	circRNA (circular RNA)	circRNA SCAR	Down-regulated in MASH; may be involved in regulating metabolic inflammation	[233]
**Monitoring Disease Progression and Treatment Response**	miRNA	miR-122	Levels decrease in response to effective treatment (reduced liver damage)	[234, 235]
	miRNA	miR-21, miR-34a	Levels decrease following weight loss or bariatric surgery	[236, 237]
	tsRNA	tRF-Val-CAC-005	Levels correlate with fibrosis progression and may predict disease progression risk	[226]

#### Pulmonary fibrosis

Idiopathic pulmonary fibrosis (IPF) is a chronic progressive interstitial lung disease. Currently, its diagnosis relies primarily on high-resolution computed tomography (HRCT) and lung biopsy, and there remains a lack of specific serological markers [238, 239]. Previous studies have shown that circulating cell-free DNA (ccfDNA) from IPF patients can be used to detect MUC5B single nucleotide polymorphisms (SNPs), and that inconsistencies exist between genotypes derived from ccfDNA and genomic DNA. To a certain extent, ccfDNA may reflect lung-specific genetic information, as ccfDNA in the blood of IPF patients is likely derived from diseased lung tissue, thus conferring a degree of disease specificity [240]. This finding suggests that liquid biopsy holds potential value in the auxiliary diagnosis, disease staging, genetic testing, and prognosis evaluation of IPF, similar to its established research paradigm in oncology.

In addition, a recent metabolomic study revealed the potential role of abnormal lipid metabolism in IPF: palmitoyl ethanolamide (PEA), along with 2-amino-1,3,4-octadecanetriol, was found to be significantly increased in the serum of IPF patients [241]. Among these, PEA has been validated as a biomarker reflecting systemic inflammatory status and the degree of lung function impairment. Additionally, 2-amino-1,3,4-octadecanetriol was the first metabolite reported to be potentially associated with an increased risk of venous thromboembolism in IPF patients [241]. These two metabolites, as novel serum biomarkers, hold promise for improving the diagnosis and prognosis of IPF. Their close correlation with multidimensional clinical parameters—including lung function, inflammatory status, coagulation state, and nutritional status—suggests they may provide new molecular targets for individualized treatment and disease monitoring.

This study further highlights the key role of lipid metabolic reprogramming in the pathogenesis of IPF and provides a new direction for the development of future treatment strategies. However, both studies have certain limitations, including small sample sizes and single-center designs, and the results require validation in large, prospective studies.

#### Lupus nephritis and renal fibrosis

Lupus nephritis (LN) is a common and serious complication of systemic lupus erythematosus (SLE). Although renal biopsy remains the gold standard for diagnosis, it is invasive, difficult to repeat, and unsuitable for dynamic monitoring. As a direct product of the kidney, urine offers the advantages of being non-invasive, repeatable, and easy to collect. Often referred to as a "liquid biopsy" of the kidney, urine represents an ideal source of biomarkers.

In recent years, scholars have systematically reviewed this field [242], encompassing cellular components (CD4^+^ T cells, Th17 cells, B cells/plasma cells) [242–245], cytokines (IFN-γ, IL-16, IL-17, TGF-β, CSF-1, TWEAK, BLyS/BAFF) [246–253], adhesion molecules (VCAM-1, ALCAM) [254–257], soluble leukocyte markers/B-cell receptors (sCD163, sCD11b, IGBP1), antibodies, light chain fragments, and complement degradation products (anti-C1q antibodies, free light chains (FLCs), C3d), proteins and small-molecule peptides (NGAL, MCP-1, KIM-1, G3BP, SerpinA3, ECM-associated peptides) [258–269], metabolomics (β-alanine, pyridoxic acid/tryptophan ratio, citrate) [270–273], and urinary exosomes and RNA (miR-29c, miR-146a, miR-26a, miR-30b, tsRNA) [248, 273–277]. From a pathological perspective, urine biomarkers can reflect LN activity, chronicity, pathological subtype, and other information, demonstrating the potential to complement or even replace renal biopsy. Although most current studies are cross-sectional and lack large-sample, prospective validation, the continued development of high-throughput technologies and omics approaches is expected to enable the establishment of standardized urine biomarker panels for early diagnosis, dynamic monitoring, and prognosis evaluation of LN in the future. Renal fibrosis is the core pathological process driving the progression of chronic kidney disease (CKD) to end-stage renal disease. Currently, renal biopsy remains the gold standard for diagnosing renal fibrosis; however, its invasive nature carries bleeding risks and potential sampling errors, limiting its widespread application. Traditional clinical indicators, such as serum creatinine and estimated glomerular filtration rate (eGFR), lack sensitivity for detecting early fibrotic changes, and the specificity of imaging examinations is also relatively limited. A recent study suggests that the expression level of growth arrest-specific 5 (GAS5) is significantly negatively correlated with the severity of renal fibrosis and demonstrates high diagnostic accuracy [278]. This finding addresses a critical gap in the non-invasive diagnosis of renal fibrosis, lays an important foundation for further multicenter and prospective clinical research, and holds promise for advancing the clinical translation of GAS5, thereby optimizing individualized management strategies for CKD patients.

#### Rheumatoid arthritis (RA)

The research and application of liquid biopsy in rheumatoid arthritis is gradually transitioning from the laboratory to the clinic. One of the core values of liquid biopsy lies in its potential to predict drug efficacy. By analyzing genetic information, epigenetic modifications, cell-free DNA (cfDNA), and microRNA (miRNA) in the blood, liquid biopsy is expected to predict patient response to traditional antirheumatic drugs such as methotrexate at an early stage of treatment [279]. This strategy contributes to the realization of "precision medicine" for RA, helping to avoid ineffective treatment and reduce unnecessary adverse drug reactions. Although epigenetic markers, cfDNA, and microRNAs show potential for predicting methotrexate response, related research is still in the early exploratory stage and lacks sufficient clinical validation (Table S1). Future large-scale, multicenter studies with standardized methodologies are needed to promote the clinical translation of these biomarkers.

For early diagnosis, traditional serological markers such as rheumatoid factor and anti-cyclic citrullinated peptide (anti-CCP) antibody have certain limitations. In contrast, liquid biopsy techniques—by detecting multiple marker types in blood, urine, and other body fluids, such as DNA, RNA, and exosomes—have opened a new pathway for the early detection of rheumatoid arthritis. Of note, a study published at the 2025 ACR Annual Meeting demonstrated that analysis of plasma using mid-infrared spectroscopy can generate disease-specific "spectral fingerprints." This technology can not only effectively distinguish between rheumatoid arthritis (RA) patients and healthy individuals, but also enable differential diagnosis of RA from other rheumatic diseases (e.g., fibromyalgia, ankylosing spondylitis). Further analysis showed that specific spectral characteristics were highly correlated with disease activity score (DAS28) and levels of inflammatory factors such as interleukin (IL)−6 and IL-17A (correlation coefficient r = 0.903). This suggests that the technology enables real-time monitoring of inflammatory burden and disease status through a simple blood test.

## Conclusions and future prospects

Liquid biopsy technology has demonstrated revolutionary potential in the diagnosis, efficacy evaluation, and dynamic monitoring of both cancerous and non-cancerous diseases. Through the discovery and application of diverse biomarkers—including circulating tumor DNA (ctDNA), circulating tumor cells (CTCs), extracellular vesicles (EVs), non-coding RNAs, and tumor-educated platelets (TEPs)—liquid biopsy is gradually transforming the traditional disease management paradigm that has relied on tissue biopsy and imaging. Substantial clinical evidence has accumulated in areas such as early cancer screening, minimal residual disease (MRD) monitoring, treatment response prediction, and analysis of tumor heterogeneity and drug resistance. At the same time, liquid biopsy has demonstrated its advantages as a non-invasive, real-time, and comprehensive diagnostic tool in non-cancer diseases, including reproductive medicine, transplant rejection monitoring, neurological disorders, and infectious diseases.

However, despite rapid technological advancements, liquid biopsy still faces multiple challenges on its path to widespread clinical application. Standardization is one of the most prominent bottlenecks, including the lack of uniformity in sample collection, processing, biomarker isolation, and detection methods, which makes it difficult to compare results across different platforms and laboratories. In addition, cost-effectiveness still needs to be optimized, particularly in low- and middle-income countries and primary care settings with limited resources. The complexity of data analysis also limits its clinical adoption. The integration and interpretation of multi-omics data require specialized bioinformatics expertise, and no standardized algorithms or clinical pathways have yet been established. Finally, the lack of large-scale prospective clinical validation means that many liquid biopsy markers remain in the research stage and have not been incorporated into routine clinical guidelines.

To address these gaps, future research should focus on the following key directions: First, promote technological standardization and automation by establishing unified standards for sample processing and testing, and developing integrated "lab-on-a-chip" systems that combine sample pretreatment with detection. This would enable "sample-to-result" one-click operation, improving both detection efficiency and reproducibility. Second, strengthen multi-omics integration and AI-assisted analysis. By fusing multidimensional data—including ctDNA, CTCs, EVs, RNA, and proteins—and applying machine learning algorithms, more accurate disease prediction and classification models can be constructed, thereby improving the precision of early diagnosis and efficacy prediction. Third, conduct large-scale, multicenter prospective clinical trials to validate the clinical utility and economic benefits of liquid biopsy. Such studies should cover diverse populations and disease stages, and explore the practical value of liquid biopsy in guiding treatment decisions and replacing traditional endpoints. For example, in cancer early screening, its contribution to mortality reduction needs to be clarified; in transplantation medicine, the specificity and sensitivity of dd-cfDNA for predicting rejection require rigorous validation. Fourth, explore novel application scenarios—such as combining liquid biopsy with spatial transcriptomics to trace CTC origins, developing targeted therapeutic strategies based on lncRNAs and TEPs, and expanding its use in psychiatric disorders and chronic inflammatory diseases—which will further broaden its clinical boundaries.

In conclusion, liquid biopsy has changed from a concept to a clinical method and enhances the scope of precision medicine. In the future, with the help of technology standardization, intelligent data processing, comprehensive clinical verification and different application fields, liquid biopsy is expected to become an important instrument throughout the whole treatment process, showing a new period of personalized, dynamic and accurate health management.

## Supplementary Information

Supplementary Material 1.

## Data Availability

Not applicable.

## Biomarkers

ctDNA: Circulating Tumor DNA
ctRNA: Circulating Tumor RNA
cfDNA: Cell-free DNA
cffDNA: Cell-free Fetal DNA
dd-cfDNA: Donor-derived cell-free DNA
mcfDNA: Microbial cell-free DNA
CTC: Circulating Tumor Cell
EV: Extracellular Vesicle
miRNA: MicroRNA
lncRNA: Long non-coding RNA
tsRNA: TRNA-derived small RNA
circRNA: Circular RNA
exRNA: Extracellular RNA
TEP: Tumor-Educated Platelet
TAA: Tumor-Associated Antigen
TAAb: Tumor-Associated Antigen autoantibody

## Detection methods and techniques

PCR: Polymerase Chain Reaction
qPCR: Quantitative PCR
ddPCR: Droplet Digital PCR
ARMS-PCR: Amplification Refractory Mutation System PCR
NGS: Next-Generation Sequencing
mNGS: Metagenomic Next-Generation Sequencing
scRNA-seq: Single-Cell RNA Sequencing
MPSS: Massively Parallel Signature Sequencing
SOLiD: Sequencing by Oligonucleotide Ligation and Detection
ELISA: Enzyme-Linked Immunosorbent Assay
Simoa: Single Molecule Array
ECL: Electrochemiluminescence
PEA: Proximity Extension Assay
FCM: Flow Cytometry
nanoFCM: Nanoscale Flow Cytometry

## Clinical concepts and applications

MRD: Minimal Residual Disease
MCED: Multi-Cancer Early Detection
NIPT: Non-Invasive Prenatal Testing
PGT-A: Preimplantation Genetic Testing for Aneuploidies
niPGT-A: Non-invasive PGT-A
bTMB: Blood Tumor Mutational Burden
bMSI: Blood Microsatellite Instability
AR: Acute Rejection
ABMR: Antibody-Mediated Rejection
ICI: Immune Checkpoint Inhibitor
NSCLC: Non-Small Cell Lung Cancer
AD: Alzheimer ‘s Disease
IPF: Idiopathic Pulmonary Fibrosis
MASLD: Metabolic dysfunction-Associated Steatotic Liver Disease
MASH: Metabolic dysfunction-Associated Steatohepatitis
NAFLD: Non-Alcoholic Fatty Liver Disease
LN: Lupus Nephritis
SLE: Systemic Lupus Erythematosus
RA: Rheumatoid Arthritis
CKD: Chronic Kidney Disease
DSA: Donor-Specific Antibody

## Other related terms

FDA: Food and Drug Administration
EpCAM: Epithelial Cell Adhesion Molecule
CK: Cytokeratin
VEGF: Vascular Endothelial Growth Factor
PDGF: Platelet-Derived Growth Factor
MAPK: Mitogen-Activated Protein Kinase
PI3K/AKT: Phosphoinositide 3-Kinase/Protein Kinase B
MAF: Mutant Allele Frequency
SNV: Single Nucleotide Variant
SNP: Single Nucleotide Polymorphism
EMT: Epithelial-Mesenchymal Transition
CCGA: Circulating Cell-free Genome Atlas
RECIST: Response Evaluation Criteria in Solid Tumors
HRCT: High-Resolution Computed Tomography
eGFR: Estimated Glomerular Filtration Rate
DAS28: Disease Activity Score 28
