# Supplementary material for: Liquid biopsy: current applications and future directions
Source: Mol Biomed. 2026 Sep 7;7:164. doi: 10.1186/s43556-026-00533-1 (PMC13550396; doi:10.1186/s43556-026-00533-1)
Supplement: Supplementary file 1 [file 43556_2026_533_MOESM1_ESM.docx]

**Supplementary Table 1. Epigenetic and Molecular Markers Associated with Methotrexate Response in Rheumatoid Arthritis**

| Categories | Subcategories | Key Findings | Clinical Relevance to MTX Response | References |
| --- | --- | --- | --- | --- |
| **Epigenetics** | DNA methylation | Global DNA hypomethylation in T cells and monocytes; MTX increases methylation and DNMT1 expression. | Methylation changes may serve as pharmacodynamic markers of MTX. | [1] |
|  |  | Methylation sites at 4 weeks post-treatment (e.g., GATA3, miR-182-related sites) correlate with 6-month efficacy. | Early methylation changes can predict long-term response. | [2] |
|  |  | Baseline ADAMTSL2 hypermethylation combined with BTN3A2 hypomethylation is positively associated with treatment response (PPV 96.6%). | Methylation profile can predict response to MTX combination therapy. | [3] |
|  |  | Baseline hypermethylation is associated with less reduction in DAS28. | Baseline methylation levels are inversely associated with response. | [4] |
|  |  | Methylation sites identified in RA-related genes, such as GATA3 and miR-182. | Methylation may influence MTX efficacy by regulating immune-related genes. | [2, 5, 6] |
|  | Citrullination/ACPA | Seropositive and seronegative patients differ in disease status and treatment response. | Results are inconsistent: some studies show better response in seropositive patients, others show no difference. | [7-11] |
|  | Acetylation | High-dose MTX inhibits HDAC and promotes acetylation. | Role of low-dose MTX in RA is unclear; no relevant studies available. | [12] |
| **Cell-free DNA** | cfDNA levels | cfDNA levels are elevated in RA patients and correlate with disease activity and tissue damage. | Relationship with MTX efficacy has not been clearly established. | [13-16] |
|  |  | cfDNA levels are high in early disease (<10 years) and may decrease in late stages. | May reflect disease stage rather than treatment response. | [14, 16] |
|  | Effect of DMARDs | Small studies show that DMARDs (including MTX) do not alter cfDNA levels. | cfDNA may not be a sensitive indicator of MTX efficacy. | [15] |
|  |  | TNF-α inhibitors can alter cfDNA levels. | cfDNA may be regulated by TNF-α and indirectly affected by MTX. | [15, 17] |
| **microRNA** | miR-16 | Targets TNF-α; in early RA patients, levels increase at 3 months and decrease at 12 months post-treatment. | Higher baseline levels associated with greater DAS28 reduction at 3 months; magnitude of change correlates with long-term efficacy. | [18] |
|  | miR-155 | Inhibits MMPs; promotes inflammatory factor expression; enhances osteoclast activity. | Responders show low baseline levels and increases at 4 months post-treatment. | [19] |
|  | miR-146a | Inhibits osteoclastogenesis and T cell apoptosis. | Responders show low baseline levels and post-treatment increases; negatively correlated with DAS28. | [17, 19, 20] |
|  | miR-132 | Targets IL-1β, IL-6, IFN-β. | Levels increase in both responders and non-responders at baseline; negatively correlated with DAS28. | [21] |
|  | miR-27a | Targets MAPK; reduces IL-6 and TNF-α; increases IL-10. | High baseline levels associated with remission at 12 months; post-treatment declines associated with remission. | [22] |
|  | miR-126-3p | Targets PIK3R2 and regulates the PI3K/AKT pathway. | Upregulated after MTX combined with anti-TNF treatment. | [23] |
|  | miR-223-3p | Targets NF-1A to regulate osteoclast differentiation. | Upregulated after MTX combined with anti-TNF treatment. | [24] |
|  | Pharmacogenetics | miR-24 regulates DHFR; DHFR SNP (C829T) is located in the miR-24 binding site; T allele abrogates miR-24 regulation. | May affect therapeutic response by regulating MTX target enzyme expression. | [25] |
|  | miRNA-methylation crosstalk | miR-301a promotes Th17 differentiation by inhibiting PIAS3; Th17 contributes to RA pathogenesis by inhibiting DNA methylation. | May serve as a pharmacodynamic marker of MTX. | [26] |

**References for Supplementary Table 1**

1.	de Andres MC, Perez-Pampin E, Calaza M, Santaclara FJ, Ortea I, Gomez-Reino JJ, Gonzalez A: Assessment of global DNA methylation in peripheral blood cell subpopulations of early rheumatoid arthritis before and after methotrexate. *Arthritis Res Ther* 2015, 17(1):233. <https://doi.org/10.1186/s13075-015-0748-5>.

2.	Nair N, Plant D, Verstappen SM, Isaacs JD, Morgan AW, Hyrich KL, Barton A, Wilson AG: Differential DNA methylation correlates with response to methotrexate in rheumatoid arthritis. *Rheumatology (Oxford)* 2020, 59(6):1364-1371. <https://doi.org/10.1093/rheumatology/kez411>.

3.	Glossop JR, Nixon NB, Emes RD, Sim J, Packham JC, Mattey DL, Farrell WE, Fryer AA: DNA methylation at diagnosis is associated with response to disease-modifying drugs in early rheumatoid arthritis. *Epigenomics* 2017, 9(4):419-428. <https://doi.org/10.2217/epi-2016-0042>.

4.	Gosselt HR, van Zelst BD, de Rotte M, Hazes JMW, de Jonge R, Heil SG: Higher baseline global leukocyte DNA methylation is associated with MTX non-response in early RA patients. *Arthritis Res Ther* 2019, 21(1):157. <https://doi.org/10.1186/s13075-019-1936-5>.

5.	Sasaki T, Onodera A, Hosokawa H, Watanabe Y, Horiuchi S, Yamashita J *et al*: Genome-Wide Gene Expression Profiling Revealed a Critical Role for GATA3 in the Maintenance of the Th2 Cell Identity. *PLoS One* 2013, 8(6):e66468. <https://doi.org/10.1371/journal.pone.0066468>.

6.	Miller CH, Smith SM, Elguindy M, Zhang T, Xiang JZ, Hu X, Ivashkiv LB, Zhao B: RBP-J-Regulated miR-182 Promotes TNF-α-Induced Osteoclastogenesis. *J Immunol* 2016, 196(12):4977-4986. <https://doi.org/10.4049/jimmunol.1502044>.

7.	Sokolove J, Schiff M, Fleischmann R, Weinblatt ME, Connolly SE, Johnsen A *et al*: Impact of baseline anti-cyclic citrullinated peptide-2 antibody concentration on efficacy outcomes following treatment with subcutaneous abatacept or adalimumab: 2-year results from the AMPLE trial. *Ann Rheum Dis* 2016, 75(4):709-714. <https://doi.org/10.1136/annrheumdis-2015-207942>.

8.	Saevarsdottir S, Wallin H, Seddighzadeh M, Ernestam S, Geborek P, Petersson IF, Bratt J, van Vollenhoven RF: Predictors of response to methotrexate in early DMARD naive rheumatoid arthritis: results from the initial open-label phase of the SWEFOT trial. *Ann Rheum Dis* 2011, 70(3):469-475. <https://doi.org/10.1136/ard.2010.139212>.

9.	Ponchel F, Goëb V, Parmar R, El-Sherbiny Y, Boissinot M, El Jawhari J *et al*: An immunological biomarker to predict MTX response in early RA. *Ann Rheum Dis* 2014, 73(11):2047-2053. <https://doi.org/10.1136/annrheumdis-2013-203566>.

10.	Ally MM, Hodkinson B, Meyer PW, Musenge E, Tintinger GR, Tikly M, Anderson R: Circulating anti-citrullinated peptide antibodies, cytokines and genotype as biomarkers of response to disease-modifying antirheumatic drug therapy in early rheumatoid arthritis. *BMC Musculoskelet Disord* 2015, 16:130. <https://doi.org/10.1186/s12891-015-0587-1>.

11.	Wevers-de Boer K, Visser K, Heimans L, Ronday HK, Molenaar E, Groenendael JH *et al*: Remission induction therapy with methotrexate and prednisone in patients with early rheumatoid and undifferentiated arthritis (the IMPROVED study). *Ann Rheum Dis* 2012, 71(9):1472-1477. <https://doi.org/10.1136/annrheumdis-2011-200736>.

12.	Yang PM, Lin JH, Huang WY, Lin YC, Yeh SH, Chen CC: Inhibition of histone deacetylase activity is a novel function of the antifolate drug methotrexate. *Biochem Biophys Res Commun* 2010, 391(3):1396-1399. <https://doi.org/10.1016/j.bbrc.2009.12.072>.

13.	Burska A, Rodríguez-Carrio J, Biesen R, Dik WA, Eloranta ML, Cavalli G *et al*: Type I interferon pathway assays in studies of rheumatic and musculoskeletal diseases: a systematic literature review informing EULAR points to consider. *RMD Open* 2023, 9(1). <https://doi.org/10.1136/rmdopen-2022-002876>.

14.	Hashimoto T, Yoshida K, Hashimoto N, Nakai A, Kaneshiro K, Suzuki K, Kawasaki Y, Shibanuma N, Hashiramoto A: Circulating cell free DNA: a marker to predict the therapeutic response for biological DMARDs in rheumatoid arthritis. *Int J Rheum Dis* 2017, 20(6):722-730. <https://doi.org/10.1111/1756-185x.12959>.

15.	Zhong XY, von Mühlenen I, Li Y, Kang A, Gupta AK, Tyndall A, Holzgreve W, Hahn S, Hasler P: Increased concentrations of antibody-bound circulatory cell-free DNA in rheumatoid arthritis. *Clin Chem* 2007, 53(9):1609-1614. <https://doi.org/10.1373/clinchem.2006.084509>.

16.	Leon SA, Ehrlich GE, Shapiro B, Labbate VA: Free DNA in the serum of rheumatoid arthritis patients. *J Rheumatol* 1977, 4(2):139-143.

17.	Bar-Yehuda S, Silverman MH, Kerns WD, Ochaion A, Cohen S, Fishman P: The anti-inflammatory effect of A3 adenosine receptor agonists: a novel targeted therapy for rheumatoid arthritis. *Expert Opin Investig Drugs* 2007, 16(10):1601-1613. <https://doi.org/10.1517/13543784.16.10.1601>.

18.	Filková M, Aradi B, Senolt L, Ospelt C, Vettori S, Mann H *et al*: Association of circulating miR-223 and miR-16 with disease activity in patients with early rheumatoid arthritis. *Ann Rheum Dis* 2014, 73(10):1898-1904. <https://doi.org/10.1136/annrheumdis-2012-202815>.

19.	Zhou Q, Haupt S, Kreuzer JT, Hammitzsch A, Proft F, Neumann C *et al*: Decreased expression of miR-146a and miR-155 contributes to an abnormal Treg phenotype in patients with rheumatoid arthritis. *Ann Rheum Dis* 2015, 74(6):1265-1274. <https://doi.org/10.1136/annrheumdis-2013-204377>.

20.	Pauley KM, Satoh M, Chan AL, Bubb MR, Reeves WH, Chan EK: Upregulated miR-146a expression in peripheral blood mononuclear cells from rheumatoid arthritis patients. *Arthritis Res Ther* 2008, 10(4):R101. <https://doi.org/10.1186/ar2493>.

21.	Singh A, Patro PS, Aggarwal A: MicroRNA-132, miR-146a, and miR-155 as potential biomarkers of methotrexate response in patients with rheumatoid arthritis. *Clin Rheumatol* 2019, 38(3):877-884. <https://doi.org/10.1007/s10067-018-4380-z>.

22.	Sode J, Krintel SB, Carlsen AL, Hetland ML, Johansen JS, Hørslev-Petersen K *et al*: Plasma MicroRNA Profiles in Patients with Early Rheumatoid Arthritis Responding to Adalimumab plus Methotrexate vs Methotrexate Alone: A Placebo-controlled Clinical Trial. *J Rheumatol* 2018, 45(1):53-61. <https://doi.org/10.3899/jrheum.170266>.

23.	Gao J, Zhou XL, Kong RN, Ji LM, He LL, Zhao DB: microRNA-126 targeting PIK3R2 promotes rheumatoid arthritis synovial fibro-blasts proliferation and resistance to apoptosis by regulating PI3K/AKT pathway. *Exp Mol Pathol* 2016, 100(1):192-198. <https://doi.org/10.1016/j.yexmp.2015.12.015>.

24.	Shibuya H, Nakasa T, Adachi N, Nagata Y, Ishikawa M, Deie M, Suzuki O, Ochi M: Overexpression of microRNA-223 in rheumatoid arthritis synovium controls osteoclast differentiation. *Mod Rheumatol* 2013, 23(4):674-685. <https://doi.org/10.1007/s10165-012-0710-1>.

25.	Mishra PJ, Humeniuk R, Mishra PJ, Longo-Sorbello GS, Banerjee D, Bertino JR: A miR-24 microRNA binding-site polymorphism in dihydrofolate reductase gene leads to methotrexate resistance. *Proc Natl Acad Sci U S A* 2007, 104(33):13513-13518. <https://doi.org/10.1073/pnas.0706217104>.

26.	Tang X, Yin K, Zhu H, Tian J, Shen D, Yi L *et al*: Correlation Between the Expression of MicroRNA-301a-3p and the Proportion of Th17 Cells in Patients with Rheumatoid Arthritis. *Inflammation* 2016, 39(2):759-767. <https://doi.org/10.1007/s10753-016-0304-8>.
